# Uncovering the Structural Landscape of Heat-Induced Riboswitch RNA Unfolding Using Native Variable-Temperature ESI Ion Mobility Mass Spectrometry and ^1^H NMR

**DOI:** 10.1021/jacsau.6c00267

**Published:** 2026-06-19

**Authors:** Sarah V. Heel-Juen, Raphael Plangger, Casey J. Chen, Zachary M. Miller, Fabian Juen, Christoph Kreutz, Evan R. Williams

**Affiliations:** † Department of Chemistry, 1438 University of California, Berkeley, California 94720-1460, United States; ‡ Institute of Organic Chemistry and Center for Molecular Biosciences Innsbruck, 27255 University of Innsbruck, Innrain 80/82, Innsbruck 6020, Austria

**Keywords:** riboswitch aptamer, RNA thermal unfolding, variable-temperature electrospray ionization (vT-ESI), ion mobility–mass spectrometry (IM–MS), charge-state distributions, native mass spectrometry, imino-proton NMR

## Abstract

RNA structures denature at elevated temperatures, leading to progressive base-pair opening and loss of secondary structure. However, the conformations adopted at solution temperatures above those at which base pairing is disrupted remain poorly understood. In this study, structural information from variable-temperature nanoelectrospray ionization ion mobility mass spectrometry, imino-proton nuclear magnetic resonance spectroscopy, and UV absorbance is combined to map the stepwise unfolding and refolding of a riboswitch aptamer. Three distinct thermal regimes were identified. In Region I (25–55 °C), the average negative charge state decreases unexpectedly and correlates with selective melting of the upper stem detected by NMR, suggesting a local reorganization that reduces the accessibility of backbone phosphate groups for deprotonation during electrospray charging. In Region II (60–75 °C), complete loss of imino resonances coincides with a sharp increase in negative charge, consistent with global loss of secondary structure and increased phosphate accessibility, enabling accommodation of higher net charge. In Region III (80–90 °C), the average charge decreases again, reflecting an increased abundance of compact conformers of the M^8–^ ion detected by ion mobility. These conformers exhibit collision cross sections up to ∼120 Å^2^ (∼10%) smaller than those of the native species. Together, these results indicate that RNA melting does not inevitably lead to complete disorder, but can produce compact, non-native conformers in which phosphate groups are partially shielded, thereby limiting charge uptake. These findings expand our understanding of RNA conformational dynamics and its capacity to adopt alternative conformations under thermal stress.

## Graphical abstract

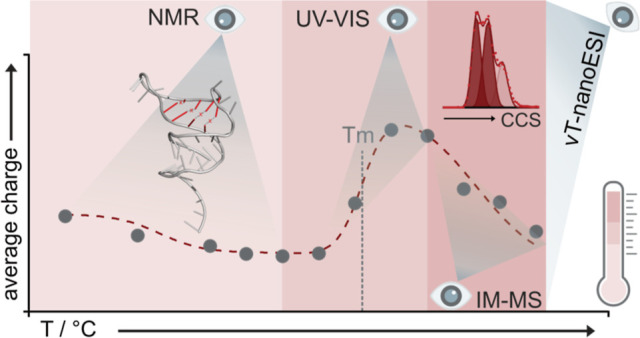

## Introduction

RNA molecules are central regulators of gene expression and cellular adaptation, and RNA structures can act as temperature-sensitive regulatory elements.^1,2^ Typical bacterial RNA thermometers regulate translation initiation via temperature-dependent rearrangements of base-pairing interactions that sequester (mask) or release (expose) the Shine–Dalgarno sequence, often together with the start codon.^3–6^ In plants and other eukaryotes, temperature-sensitive RNA structures in untranslated regions can modulate transcript stability and translation efficiency,^1,7,8^ while temperature-dependent alternative splicing of stress-related pre-mRNAs provides an additional regulatory layer.^1,9,10^ Together, these mechanisms indicate that heat-induced RNA structural changes are not merely stochastic degradation events but regulated processes with physiological relevance across kingdoms of life.^1,2,4,5^ In a broader perspective, understanding how RNA responds to elevated temperatures is also relevant for predicting the resilience of gene-regulatory systems under environmental stress, including the increasing frequency and intensity of heatwaves associated with climate change.^11–13^

RNA structures are stabilized by base pairing, stacking interactions, ion coordination, and increasing temperature progressively disrupts these interactions.^14–18^ Unlike DNA duplexes, which melt into separated strands, RNA-being single-stranded and highly flexible-undergoes a stepwise unfolding process that may populate non-native conformers.^19^ Conventional spectroscopic methods, such as UV absorbance and circular dichroism, primarily report on base-pair disruption and the loss of stacking interactions and therefore capture the global loss of secondary structure.^20–22^ However, they provide limited information about conformational heterogeneity, particularly above the melting temperature where native base pairing is largely lost and the spectra reflect ensemble-averaged stacking interactions. It therefore remains unclear whether thermally unfolded RNAs exist solely as extended, disordered chains or whether alternative non-native conformers can also be adopted under such conditions.

Variable-temperature electrospray ionization (vT-ESI) has recently emerged as a powerful approach to probe biomolecular unfolding in solution prior to transfer into the gas phase.^23,24^ For proteins, vT-ESI coupled with ion mobility mass spectrometry (IM–MS) has been used to track heat-induced expansion and compaction with conformer-level resolution.^25–27^ More recently, temperature-controlled nanoelectrospray ionization coupled with IM–MS has enabled thermodynamic analysis of higher-order DNA structures, including three-way junctions.^28^ These studies demonstrate that vT-ESI—particularly when combined with ion mobility—can resolve temperature-induced structural transitions in both proteins and DNA.^25,26,28^

By comparison with proteins and double-stranded DNA, RNA presents a greater challenge. RNA is not only more conformationally dynamic, but it also lacks the clear strand-separation signature characteristic of DNA melting.^14,19,29^ Instead, RNA often unfolds through multiple, partially overlapping transitions that generate heterogeneous conformational ensembles.^16,18^ Capturing this complexity therefore requires complementary methods that can resolve both global structural changes and local base-pair disruptions.

To address this gap, we investigated the thermal unfolding of a riboswitch aptamer using variable-temperature nanoelectrospray ionization ion mobility mass spectrometry (vT-nanoESI IM–MS), ^1^H NMR spectroscopy, and UV absorbance. This aptamer provides an ideal model system because its architecture comprises multiple discrete secondary-structure elements—a GC-rich and thermodynamically stable lower stem, an upper stem interrupted by an internal loop, and a terminal hairpin loop—that can be selectively destabilized across different temperature regimes. Specifically, we studied the 40 nt aptamer domain of a neomycin-responsive riboswitch derived from the engineered M4 variant with the highest regulatory activity.^30–32^ Our data show that, rather than denaturing into fully extended coils, the riboswitch can reorganize into unexpectedly compact, base pair–free conformers with reduced phosphate exposure relative to the ensemble observed at the melting temperature. These findings provide a new perspective on RNA conformational dynamics under heat stress and highlight the capacity of RNA to adopt non-native compact states even under extreme conditions.

## Experimental Section

Ion mobility–mass spectrometry (IM–MS) experiments were carried out on a Synapt G2-Si quadrupole time-of-flight instrument (Waters Corporation, Milford, MA, USA) operated in negative ion mode under native-like conditions.^25^ Source parameters were optimized to minimize activation: sampling cone voltage 30 V, source offset 15 V, and source temperature 85 °C. The gas pressures were maintained at ∼0.03 mbar (nitrogen) in the trap cell, ∼4.2 mbar (helium) in the helium cell, and ∼3.2 mbar (nitrogen) in the ion mobility cell. Drift time calibration was performed using protein standards of known mass and collisional cross-section—insulin, lysozyme, ribonuclease A, cytochrome *c*, and α-lactalbumin—each dissolved in 100 mM ammonium acetate (pH 6.9). The calibration procedure followed the approach described by Bush et al. and Thalassinos et al.^33,34^ Collision cross sections (CCS) were obtained by fitting measured drift times to a calibration curve (Supporting Information Figure S3). All data were processed in MassLynx v4.1 (Waters Corporation). Arrival-time distributions were fitted in Igor Pro (WaveMetrics) using Gaussian models. For the M^8–^ ion, distributions were modeled as the sum of three Gaussian components to quantify overlapping conformer populations. Fit quality was assessed from residuals, and σ denotes the root-mean-square (RMS) of the residuals reported by Igor Pro (Fit Wave Statistics). Two-dimensional heat maps depicting ion mobility intensity as a function of CCS and solution temperature were generated from raw arrival time distribution (ATD) data using a custom Python script. Drift times were converted to CCS values using a linear calibration curve (Figure S3) as described above. For each charge state and temperature point, ATDs from up to three independent runs were normalized to 100% relative intensity, interpolated onto a uniform CCS grid (800 points) using cubic spline interpolation,^35^ and averaged across valid runs. The averaged profiles were linearly interpolated onto a uniform temperature grid (25–90 °C) and rendered using the inferno colormap (matplotlib).^36^ For the M^17–^ ion, variable-temperature ion mobility data were too sparsely populated across the temperature range to allow reliable interpolation; no heatmap was therefore generated for this charge state. CIU heat maps were generated using the same Python script with collision energy instead of temperature on the *x*-axis (CE = 0–45 V). Data at CE = 50 V are excluded as extensive fragmentation at this collision energy results in insufficient precursor signal-to-noise for ion mobility analysis (Figure S5). CIU heat maps are shown for the M^8–^ ion recorded with and without quadrupole isolation.

Variable-temperature nanoelectrospray ionization (vT-nanoESI) was performed using a custom-built resistively heated source based on the design of Sterling and Williams.^25,37^ The aluminum heating jacket was wrapped with nichrome wire and controlled via an Omega CNi-3222 temperature controller (Stamford, CT, USA).^25^ K-type thermocouples monitored both the jacket temperature and, during calibration, the temperature of the solution near the emitter tip.^25^ To validate the performance of the vT-nanoESI apparatus, we measured the heat-induced unfolding of cytochrome c, a well-characterized model protein. The melting temperature of cytochrome c was determined by fitting the intensity-weighted average charge state^50^ from individual ESI spectra acquired across a temperature range of 25 to 90 °C to a two-state model (see Supporting Information Figure S1).^25^ A melting temperature of 72.7 ± 0.2 °C was obtained, consistent with previously reported values,^25,38–41^ thereby confirming that our vT-nanoESI apparatus reliably monitors temperature-dependent structural changes in solution. Target temperatures were set in a stepwise temperature ramp from 25 to 90 °C for all melting experiments (cytochrome *c* and RNA), with each temperature step equilibrated for ∼60 s before data acquisition. Spectra were acquired for 1–3.5 min per temperature step. Total evaporation over the course of a full temperature ramp was determined gravimetrically by weighing nanoESI emitters empty, loaded, and after a complete temperature ramp (25–90 °C, 12 temperature steps). Each temperature step was held for 2.5 min to account for the total time spent at each temperature during a typical experiment, including equilibration and data acquisition. Approximately 30% of the initial volume (∼3 μL from an initial volume of ∼10.6 μL) was lost over the course of the ramp, leaving ∼70% (∼7.6 μL), which was sufficient to maintain stable electrospray and continuous platinum wire contact throughout data acquisition. Glass capillaries were pulled to tip inner diameters of 1.3 ± 0.1 μm or 0.70 ± 0.1 μm using a P-87 Flaming/Brown micropipette puller (Sutter Instruments, Novato, CA, USA), with geometries confirmed by scanning electron microscopy (Hitachi TM-1000).^42^ Negative nanoESI was initiated by applying −0.70 to −1.2 kV via a platinum wire inserted into the sample solution. Two of the experiments were conducted using nanoESI emitters with an inner diameter of 1.3 μm, in which the temperature of the ESI solution was gradually increased from 25 to 90 °C. In the third experiment, smaller diameter (700 nm) emitters were used to investigate the potential impact of droplet lifetime on RNA unfolding. However, nanoESI at elevated temperatures near the boiling point of water (80–90 °C) is particularly challenging, as bubble formation can cause spray instability, especially when using smaller emitter diameters such as 700 nm. To address this, the third experiment was conducted using two separate 700 nm emitters: the first nine temperature points (25–75 °C) were recorded using one emitter, while the high-temperature points (80, 85, and 90 °C) were acquired with a second emitter to minimize spray duration and maintain stability.

The neomycin-sensing riboswitch aptamer was synthesized by solid-phase phosphoramidite chemistry using established protocols.^43,44^ Crude RNA was purified by high-performance liquid chromatography (HPLC) and desalted using Vivaspin 500 centrifugal concentrators (3 kDa MWCO; Vivaproducts Inc., Littleton, MA, USA) as previously described.^32,43,44^ For all IM–MS experiments, RNA was annealed by heating to 90 °C for 2 min followed by rapid cooling on ice, then diluted to 5 μM in 100 mM ammonium acetate. Solution pH was measured prior to each experiment using a calibrated pH meter (pH 6.89). Since the small sample volume remaining at the end of a full temperature ramp does not allow direct pH measurement after the experiment, pH stability was assessed by evaporating ∼30% of a 20 μL 100 mM ammonium acetate solution (pH 6.89), consistent with the total evaporation determined gravimetrically, yielding a pH of 6.75. This confirms that buffer concentration upon evaporation does not significantly alter solution pH. Furthermore, experiments were conducted in negative ionization mode over ∼30 min, under which ESI-induced pH drift is substantially smaller than in positive ionization mode and occurs in the opposite direction, tending to slightly increase pH.^45^ The two effects are therefore expected to partially offset each other, and we conclude that the solution pH remained close to 6.9 throughout these experiments.

For NMR experiments, RNA was dissolved to a final concentration of 300–500 μM in 90 mM ammonium acetate buffer (pH 6.8) containing 10% D_2_O. Spectra were acquired at 25–65 °C on a Bruker Avance 4 Neo 700 MHz spectrometer equipped with a Prodigy TCI cryoprobe. Imino proton resonances were monitored using a 1D ^1^H selective excitation experiment and jump-and-return 2D NOESY experiments. Data were processed in Topspin 4.2.0, and resonance assignments were performed using POKY software.

## Results and Discussion

### Average Charge & ^1^H NMR

Native mass spectrometry can reveal changes in molecular conformation in solution both from changes in charge-state distributions and from changes in ion collisional cross sections measured with ion mobility.^23–28^ For proteins, denaturation leads to a substantial increase in average charge, attributed to increased solvent exposure of ionizable sites and reduced Coulombic repulsion.^25,46–49^ For RNA, deprotonation of the phosphate backbone serves as the primary origin of net charge, such that changes in average charge reflect conformational rearrangements that modulate phosphate-site accessibility during ESI.

To determine how RNA charge-state distributions depend on solution temperature, temperature-dependent nanoESI mass spectra of the riboswitch aptamer were acquired (Figure 1A). The average charge state as a function of temperature, calculated as the abundance-weighted mean across all observed charge states,^50^ is shown in Figure 1B. In contrast to the two-state unfolding transition observed for proteins such as cytochrome c (Figure S1)^25,26^ where the temperature-dependent average charge state data can be fit to a sigmoidal curve with a sharp transition corresponding to unfolding, the RNA exhibited a more complex, triphasic thermal behavior (Figure 1B).

**Figure 1:**
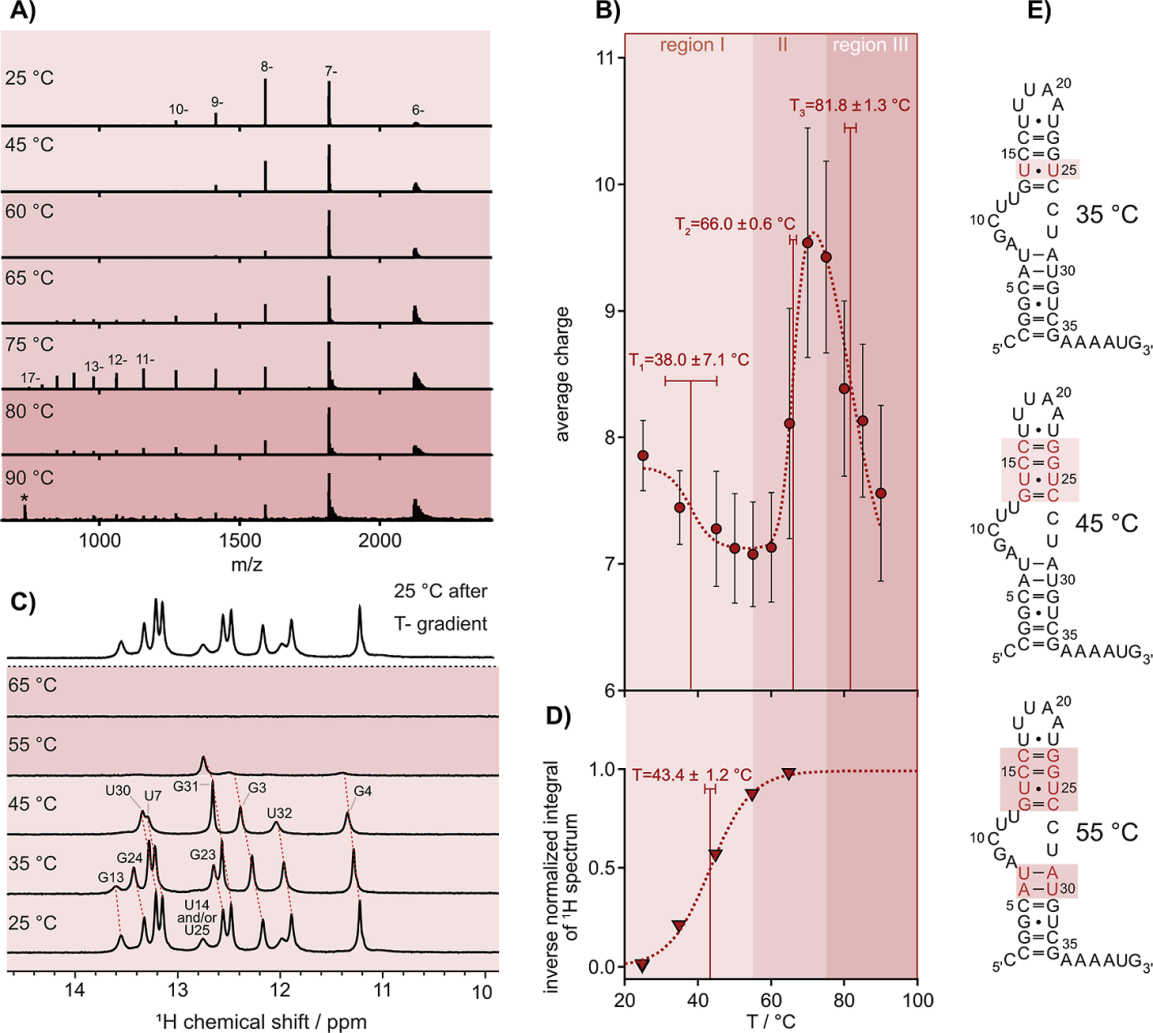
Effect of temperature on RNA structure revealed by nanoESI charge state distributions and temperature-dependent ^1^H NMR spectra. (A) Native nanoESI mass spectra of 5 μM RNA in 100 mM ammonium acetate acquired at increasing solution temperatures. Adducts observed for lower charge states are nonspecific NH_4_^+^ and Na^+^ adduction to the phosphate backbone. The asterisk (*) in the spectrum at 90 °C marks an unassigned peak (for more details see Figure S4). (B) Average charge states as a function of temperature obtained from nanoESI spectra. Each data point represents the mean of three independent experiments, with error bars indicating the standard deviation. A triple-sigmoid fit (red dashed line) reveals three distinct transitions with fitted transition temperatures *T*_1_, *T*_2_, and *T*_3_ (±standard errors from the nonlinear fit), highlighted along a red color gradient: light red (Region I: 25–55 °C), medium red (Region II: 60–75 °C), and dark red (Region III: 80–90 °C). (C) ^1^H NMR spectra in the imino proton region (RNA in ammonium acetate buffer with 10% D_2_O) recorded at various temperatures. Individual imino proton signals are assigned to specific nucleobases based on previous work.^32^ (D) Inverse normalized integral of individual imino proton signals as a function of temperature, fitted with a sigmoid function (red dashed line). The extracted melting temperature and error are derived from the fit parameters. (E) Secondary structure of the RNA riboswitch. Nucleobases shown in red correspond to residues for which imino proton signals are absent at the indicated temperatures (35 °C, 45 °C, and 55 °C), consistent with local base pair melting.

In three replicate measurements using emitters with two different tip diameters, the average charge as a function of temperature consistently revealed three well-defined transitions across all three experiments, corresponding to three distinct temperature ranges: Region I (25–55 °C), Region II (60–75 °C), and Region III (80–90 °C), highlighted by a red color gradient from light (Region I) to dark (Region III) in Figure 1B. At 25 °C in Region I, the average charge is 7.9 ± 0.3. There is a slight decrease in average charge state with increasing temperature in this Region. In Region II, the average charge state rises with temperature to a maximum of 9.5 ± 0.9 at 70 °C, before decreasing again to 7.6 ± 0.7 at 90 °C in Region III. Error bars are largest at higher temperatures where the average charge changes with temperature more significantly. Small run-to-run differences in effective solution temperature and associated spray conditions (e.g., tip positioning, emitter diameter, and droplet size) can lead to noticeable shifts in charge leading to larger measurement uncertainty. This is consistent with the high temperature sensitivity of RNA secondary structures, for example, RNA thermometers have been reported to regulate translation with sensitivities down to ∼1 °C in some systems.^2,5^ Nevertheless, the triphasic profile was reproduced consistently across all three measurements, indicating that the observed transitions reflect robust temperature-induced structural rearrangements rather than uncontrolled variation in electrospray conditions.

Since RNA melting is primarily governed by base pairing interactions that define its secondary structure,^16,18,20^ we hypothesized that each of the identified transitions corresponds to the progressive disruption of key elements essential for RNA secondary structure. To better understand the structural changes that produce these variations in average charge as a function of temperature (Figure 1B), temperature-dependent ^1^H NMR measurements were performed. Imino protons are a reliable indicator of RNA base pairing because they participate in hydrogen bonding and are protected from rapid solvent exchange. When base pairs melt, these signals disappear due to increased proton exchange with the solvent,^16,18^ providing a sensitive probe of local structural changes and residue-specific insight into the RNA unfolding pathway. Figure 1C shows temperature-dependent spectra from these experiments.

At 25 °C, the ^1^H NMR spectrum exhibits distinct imino proton resonances for all the expected canonical and noncanonical base pairs, except for the U17·U22 wobble pair (Figure 1C) located next to the hairpin loop (Figure 1E).^32^ The absence of this signal likely reflects increased local dynamics in this flexible region and only a small degree of stabilizing stacking interactions. Overall, the resonance pattern confirms that the riboswitch aptamer adopts the predicted secondary structure, with both the upper and lower stems fully base-paired.^30–32^ This complete set of imino proton signals was observed in ammonium acetate buffer under near-native conditions closely matching those used for MS experiments, demonstrating that these conditions support expected folding of the RNA secondary structure.

Individual imino proton signals decrease with temperature to different extents. However, all signals are lost at 65 °C, indicating that the hydrogen bonding interactions that govern RNA secondary structure no longer exist in a high population. The integrated signal as a function of temperature is shown in Figure 1D. A sigmoidal fit yields a transition temperature of 43.4 ± 1.2 °C, corresponding to the midpoint of upper stem destabilization.

To dissect the unfolding behavior in more detail, we next examined each of the three temperature regions individually, correlating the disappearance of imino proton signals with temperature-dependent shifts in average charge to directly link local base-pair disruptions to global structural changes in RNA. Below, we describe the unfolding events underlying Regions I-III and propose a stepwise unfolding model for the thermal denaturation pathway of the riboswitch aptamer.

### Region I (25–55 °C): Unfolding of the Upper Stem Region

In Region I (25–55 °C), the average charge decreases modestly but reproducibly, reflected by reduced abundances of higher charge states (M^10–^, M^9–^, and M^8–^) and a corresponding increase in lower charge states (e.g., M^6–^) (Figure 1A-B). This decrease in average charge in temperature Region I is unexpected because protein unfolding typically leads to an increase in charge. However, the ^1^H NMR spectra provide a structural rationale for this trend in charging. Even with a small temperature change to 35 °C, the imino proton signal for U14/U25 disappears, indicating that the U25·U14 wobble base pair-part of the upper stem-is the first to melt (Figure 1C). At 45 °C, additional imino proton signals from neighboring G = C base pairs (G24 = C15, G23 = C14, G13 = C26) also vanish, demonstrating that the upper stem is fully disrupted and is no longer base paired (Figure 1C). The resulting loss of stabilizing base-pair interactions may lead to a partial collapse or structural reorganization of the riboswitch aptamer in the upper stem region, in which phosphate groups become less prone to deprotonation. This hypothesis provides a plausible explanation for the initial drop in average charge. The first transition of the triple sigmoid fit applied to the average charge data in Figure 1B corresponds to Region I and yields a transition temperature of 38.0 ± 7.1 °C (uncertainty = standard error from the nonlinear fit), which likely reflects the melting point of the upper stem. This value is in good agreement with the ^1^H NMR data, which show complete loss of base pairing in the upper stem region at 45 °C. Interestingly, this decrease in average charge plateaus around 45 °C and remains stable up to 60 °C, indicating that the disruption of base pairs in the lower stem at 55 °C (U7-A29, U30-A6, Figure 1C) does not significantly affect the overall charge states of the RNA molecules. One possible explanation is that the three remaining base pairs in the lower stem (G3 = C32, G4·U32, G31 = C5), whose imino proton signals persist until 65 °C, are stable enough to maintain the structural integrity of the lower stem (Figure 1C).

### Region II (60–75 °C): Complete Loss of Secondary Structure

In Region II (60–75 °C), there is a sharp increase in average charge, indicating a major structural transition toward an unfolded RNA conformation (Figure 1B). ESI-MS spectra show the emergence of highly charged ions with up to 17 negative charges, indicating significant exposure of the RNA’s phosphate backbone (Figure 1A). ^1^H NMR spectra acquired in this temperature range show the complete disappearance of all imino proton signals, including those from the lower stem (G3 = C32, G4·U32, G31 = C5) (Figure 1C). Both the average charge state and the NMR data suggest that all secondary structure elements stabilized by base pairing, including both stems of the riboswitch aptamer, melt completely within this temperature range. The strong correlation between the loss of secondary structure and the steep increase in negative charge of RNA ions supports the idea that global unfolding results in extensive phosphate deshielding, which in turn enables increased charge accommodation during electrospray ionization. However, complete loss of base pairing does not necessarily imply a fully extended, disordered conformation, as residual structural elements such as transient stacking interactions or backbone organization may persist and partially shield phosphate groups in a subset of molecules, consistent with the presence of low charge states in this temperature region. The second transition of the triple sigmoid fit applied to the average charge data in Figure 1B corresponding to Region II yields a transition temperature of 66.0 ± 0.6 °C. This value closely matches the RNA melting temperature of ∼62 °C determined by absorbance at 260 and 250 nm (Figure S2). Both the NMR data and the UV data indicate that the base pair interactions are disrupted in this temperature range. The excellent agreement between the NMR, UV absorbance, and ESI MS data indicates that vT-nanoESI is not only suitable for determining protein melting temperatures but also serves as a valuable method for assessing RNA thermal stability.

### Region III (80–90 °C): Heat-Induced Collapse into a Compact Non-Native Conformer

The third transition observed in the average charge data between 80 and 90 °C is marked by a rapid decrease in charge (Figure 1B), indicating that the RNA adopts a more compact conformation. This is reflected in the charge-state distributions, with a reduction in highly charged species (M^17–^ to M^9–^) and an increase in lower charge states, such as M^6–^ (Figure 1A). This rearrangement occurs at very high temperatures despite the absence of base pairing and stacking interactions, as confirmed by the complete loss of imino proton signals above 65 °C (Figure 1C) and the absence of additional transitions in the UV melting curve between 80 °C and 90 °C (Figure S2). Thus, the compaction does not reflect renewed base pairing or stacking interactions but instead may arise from backbone-centered rearrangements that shield phosphate groups and thereby lower the net charge of the RNA ions in the gas phase. A plausible scenario is that, at these elevated temperatures, nucleobases rotate outward into solvent while the sugar–phosphate backbone folds inward, producing a compact, non-native conformer with reduced charge. Compaction that reduces hydration and solvent accessibility can also decrease charging, as observed for cytochrome c where a compact low-charge subpopulation persists (and can increase) at elevated solution temperature alongside highly charged unfolded ions.^25^

Neither NMR nor UV absorbance provide information about the nature of the high-temperature non-native structures. Both methods primarily report on nucleobase interactions, namely hydrogen bonding (NMR)^18^ and base stacking (UV absorbance).^20^ By contrast, vT-nanoESI is uniquely sensitive to changes in phosphate exposure, making it possible to resolve compact, non-native structures. These findings emphasize the strength of vT-nanoESI MS in probing backbone-centered conformational changes at elevated temperatures, complementing classical solution-based approaches.

### Ion Mobility Reveals Temperature-induced Structural Dynamics of Distinct Charge States

To gain deeper insight into the conformational landscape of the riboswitch aptamer during thermal unfolding, temperature-resolved ion mobility mass spectrometry (IM–MS) measurements were made across the full range of observable charge states. Ion mobility separates ions based on their charges and collision cross sections (CCS). The latter can provide some useful information about an ion’s size and shape in the gas phase that can reflect solution-phase structure.^34,51–53^ Distinct conformers with the same *m*/*z* can be separated by IM-MS making it possible to detect temperature-induced structural changes that occur in solution and that persist after transfer of the molecules from solution into the gas phase.^25,26^

There were minimal changes in CCS values for most ions (M^17–^ to M^6–^) over the entire temperature range (25–90 °C), with average CCS values remaining constant within ≤10 Å^2^ for a given charge state, corresponding to relative variation of only 0.3–0.8% (Figure 2A). A linear fit analysis of CCS versus temperature further confirmed this finding: for all charge states except M^8–^ and M^9–^, the slopes were within ±0.1 Å^2^/°C (Figure 2A-B). This indicates that the CCS values of the majority of ions remain constant across the entire temperature range. The arrival time distributions for these ions were Gaussian, with no substantial change in peak position, shape, or width (Figures S10–S14), indicating that either no significant measurable structural change occurred or any structural changes that did occur were not reflected by these charge states. Variable-temperature ion mobility heatmaps displaying relative intensity as a function of CCS and solution temperature for all charge states (M^6–^ to M^17–^) are provided in Figures S10–S14. Since charge-state distributions reflect solution conformation, substantial solution-phase structural changes can manifest as shifts in the relative abundances of charge states, but not necessarily as changes in the ion mobility profiles (or CCS values) of individual charge states.^27,54^

**Figure 2:**
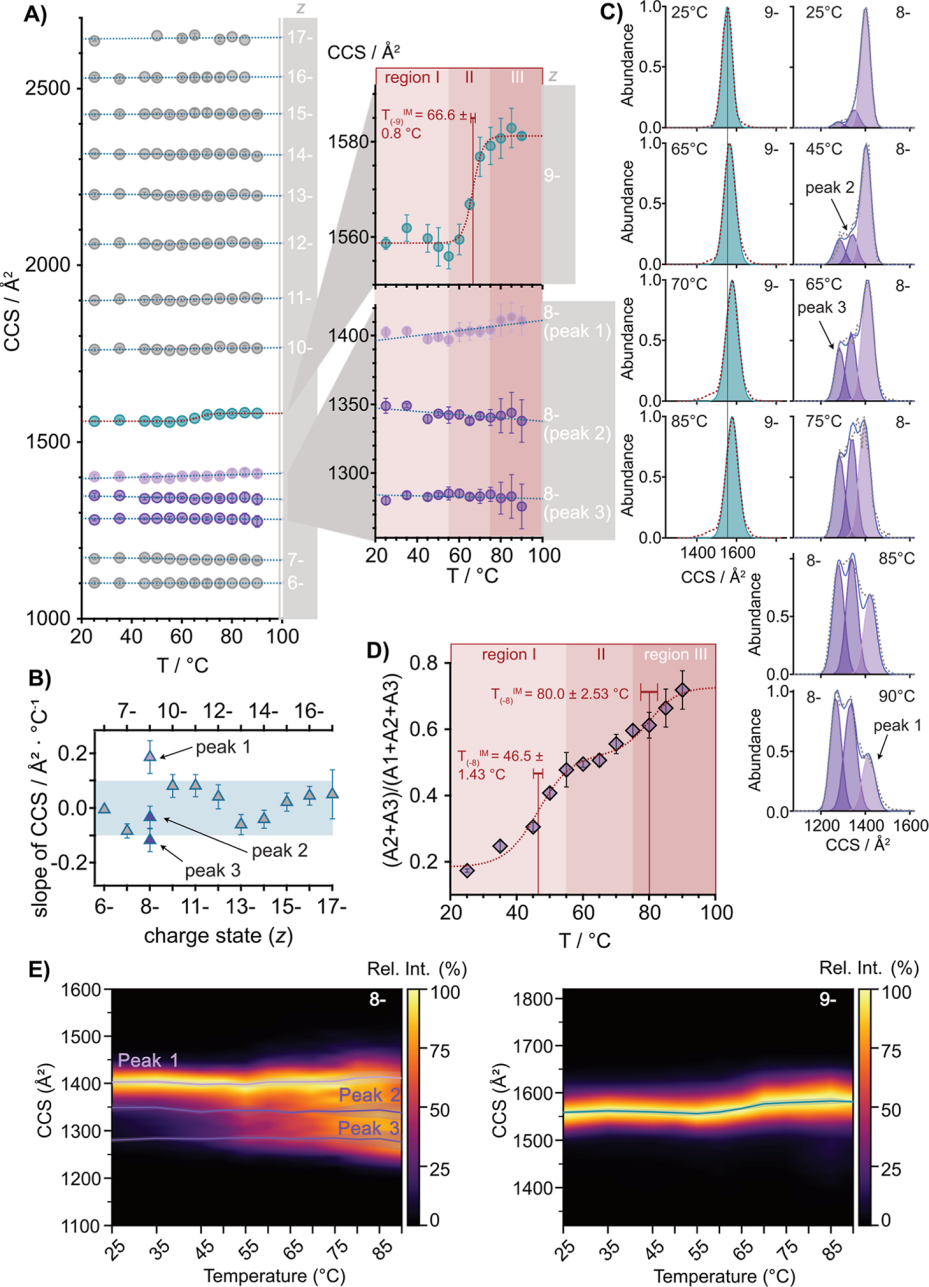
Collision cross section (CCS) analysis as a function of temperature for individual charge states (*z*). (A) Overview of CCS values between 25 and 90 °C for ions with *z* = −6 to −17. Each point is the mean of three independent experiments; error bars are standard deviations. Blue dashed lines show linear fits for all charge states; for the M^9–^ ion a sigmoid fit (red dashed line) was used because a linear model did not capture the temperature dependence. The right panel is an *x*-axis zoom highlighting the two charge states that change with temperature: M^8–^ (purple) and M^9–^ (turquoise). A light/mid/dark red background gradient indicates temperature Regions I (25–55 °C), II (60–75 °C), and III (80–90 °C), consistent with Figure 1. (B) Slope analysis derived from panel A showing negligible temperature dependence for most charge states (|slope| ≤ 0.1 Å^2^ °C^1–^) except M^8–^. The M^9–^ ion is not included in this analysis as its CCS values display a sigmoidal temperature dependence. (C) CCS distributions for M^8–^ (purple) and M^9–^ (turquoise) at selected temperatures. Dashed lines: experimental data; solid lines: Gaussian fits. For M^8–^, three conformers are resolved by triple-Gaussian fitting. Peak 1 (light purple, native-like) decreases in relative abundance with temperature, while the compact conformers (peaks 2 and 3, dark purple) increase. (D) Ratio of the combined peak areas of conformers 2 and 3 to the sum of all peak areas, (A_2_ + A_3_)/(A_1_ + A_2_ + A_3_), versus temperature. Points are means of three experiments; error bars are standard deviations. A double-sigmoid fit (red dashed line) reveals two transitions corresponding to Region I (25–55 °C; light red) and Region III (80–90 °C; dark red). (E) Variable-temperature ion mobility heat maps for the M^8–^ ion (left) and M^9–^ ion (right). Data represent the average of 3 independent measurements. Relative intensity is shown as a function of CCS and temperature (25–90 °C), normalized to 100% per temperature step. Overlaid lines indicate the mean CCS values from panel A, with shaded areas representing the corresponding standard deviations. The gradual broadening and shift of the intensity distribution at elevated temperatures reflects the conformational transitions described in panels A–D.

Interestingly, two intermediate charge states displayed clear and reproducible trends in their collisional cross sections with temperature. M^9–^ exhibited a pronounced thermally induced structural expansion, with CCS values increasing by ∼30 Å^2^ between 60 and 75 °C before reaching a plateau at higher temperatures (Figure 2A,C and E, highlighted in turquoise). This expansion coincides with the sharp increase in average net charge and the complete loss of imino proton resonances observed in ^1^H NMR (Figure 1), consistent with global melting of RNA secondary structure in solution. A sigmoidal fit of the CCS values for M^9–^ yielded a transition temperature of 66.6 ± 0.8 °C, which is in excellent agreement with the second transition temperature derived from average charge analysis (66.0 ± 0.6 °C; Figure 1B) and closely matches the melting temperature obtained from UV absorbance measurements (∼62 °C; Figure S2).

The M^8–^ showed the opposite trend: pronounced heat-induced compaction. At 25 °C, the majority of the population corresponds to a single dominant conformer (Figure 2B, peak 1), with minor abundances of more compact species indicated by a tailing of the peak toward lower CCS values. The abundances of these compact conformers increase with temperature, giving rise to two additional, albeit unresolved, peaks (peaks 2 and 3, Figure 2C). Peaks 2 and 3 correspond to the heat-induced compact states, while peak 1 corresponds to the dominant conformer at 25 °C (Figure 2C).

Each CCS distribution was modeled as the sum of three Gaussian functions representing peaks 1–3. Peak centers and widths were first determined from spectra at higher temperatures (≥80 °C), where the conformers were best resolved, and these parameters were then used as reference values at lower temperatures. For temperatures <80 °C, peak widths were constrained to within ±25% of the high-temperature reference to stabilize the fits when the two compact conformers were only partially resolved. All fits converged across the entire temperature range. Fit quality was assessed from residual plots (data-fit) and by the root-mean-square of the residuals (RMS, σ) reported by Igor Pro (Fit Wave Statistics). Residuals showed no systematic structure, indicating that the triple-Gaussian model captured the experimental ion-mobility distributions across the full temperature range (Figures S7–S9). The RMS was on the order of 10^–2^ in normalized intensity and corresponded to deviations of at most ∼1.4% of the normalized peak intensity (Figures S7–S9). Parameter precision was evaluated from the fitted uncertainties: peak centers (*x*_0_, reported as CCS in Å^2^) had standard uncertainties typically ∼0.9–3.6 Å^2^ (maximum ∼7.3 Å^2^), and peak widths had uncertainties of ∼0.6–4.5 Å^2^ (maximum ∼7.3 Å^2^). These uncertainties were within the variability observed across triplicate measurements, supporting our conclusion that the deconvolution reproducibly resolves three conformers over the full temperature range. In Regions I and II (25–75 °C), the CCS values of all three conformers remain comparatively stable, showing no significant temperature-dependent changes (Figure 2A, right). On average, across the temperature range of 25–75 °C and over three independent runs, peak 2 is 58.5 ± 4.2 Å^2^ smaller and peak 3 is 117.8 ± 3.4 Å^2^ smaller than the major conformer at 25 °C (Figure 2A, right). In Region III (80–90 °C), however, peaks 2 and 3 shift toward progressively larger CCS values. Across this high-temperature range, their differences relative to peak 1 increase to 70.7 ± 2.0 Å^2^ for peak 2 and 131.6 ± 3.0 Å^2^ for peak 3, indicative of a slight thermal expansion (Figure 2A, right). While CCS values provide direct evidence for heat-induced compaction, the relative contribution of the compact conformers to the overall ensemble was further quantified by determining the ratio of their peak areas (A_2_ + A_3_) to the sum of all peak areas (A_1_ + A_2_ + A_3_) as a function of temperature (Figure 2D). This analysis revealed two well-defined transitions (Figure 2D). In Region I, the first transition occurs at 46.5 ± 1.4 °C and reaches a plateau at ∼55 °C, where the ratio (A_2_ + A_3_)/(A_1_ + A_2_ + A_3_) stabilizes at ∼0.5 (Figure 2D). This indicates that approximately half of the population has converted into compact conformers, while the other half remains in the initial dominant state (peak 1). This transition closely coincides with the first transition temperature derived from average charge analysis (38.0 ± 7.1 °C, Figure 1B) and the melting temperature obtained from the NMR integral of imino proton resonances (43.4 ± 1.2 °C, Figure 1D), which represents the melting of the upper stem. In Region III, the second transition is observed at 80.0 ± 2.5 °C (Figure 2D), in excellent agreement with the high-temperature transition obtained from average charge data analysis (81.8 ± 1.3 °C, Figure 1B). At these elevated temperatures, the compact conformers dominate, accounting for ∼0.7 of the total peak area (Figure 2D). Consequently, the conformer initially most abundant at 25 °C (peak 1) becomes a minor species at high temperatures, representing only about one-third of the ensemble. This mirrors the decrease in average net charge observed in Region III (Figure 1B), where fully unfolded RNA reorganizes into a compact, base-pair-free ensemble that shields portions of the phosphate backbone despite lacking secondary structure. Notably, the error bars are larger in the transition regions, particularly at 55 °C and 80–90 °C. This variability in the IM data in the transition regions is also likely due to small run-to-run differences in temperature, tip positioning and droplet size.

Both M^8–^ and M^9–^ ions are required to fully capture the three distinct temperature transitions: melting of the upper stem (Region I), global unfolding of the RNA (Region II), and high-temperature structural compaction (Region III), the latter closely matching the transitions observed by average charge analysis. The close agreement of the transition temperatures obtained by either average charge analysis or ion mobility of M^8–^ and M^9–^ underscores that RNA melting events can be monitored from complementary perspectives—either at the level of the global charge distribution of the molecule or by individual ions that preserve structural features in the gas phase while remaining sensitive to heat-induced structural changes.

Taken together, not all charge states are equally informative for probing native structural features of oligonucleotides by ion mobility. Intermediate charge states can be particularly well suited for obtaining structurally informative measurements, where the charge is sufficient to produce measurable CCS differences between conformers, yet low enough to better preserve native intramolecular interactions, as has been demonstrated for both proteins and nucleic acids.^25,26,52,53,55,56^ For the riboswitch aptamer studied here, the M^8–^ ion (charge/phosphate ratio = 0.21) yielded the most pronounced and well-resolved temperature-induced conformational changes, behaving analogously to the structurally sensitive intermediate charge states (charge/phosphate ratio 0.27–0.30) identified by Benabou et al. for DNA i-motif structures.^52^ By contrast, the M^9–^ ion, with slightly higher charge density, exhibited heat-induced expansion rather than compaction, consistent with stronger Coulomb repulsion driving more extended conformations. These results highlight that intermediate charge states are particularly well suited for resolving subtle structural features of oligonucleotides by ion mobility.

### Collision-Induced Unfolding of the M^8–^ Ion

The structural compaction reflected by the reduced CCS values of M^8–^ at elevated solution temperatures provides strong evidence that this behavior originates in solution rather than the gas phase. To exclude the possibility that the observed compaction results from gas-phase rearrangements, collision-induced unfolding experiments were performed.^57,58^ In these experiments, the collision energy was incrementally increased from 0 to 50 V while monitoring changes in the arrival time distributions at a constant solution temperature of 25 °C.

At 10 V, the relative abundances of the three partially resolved conformers remained unchanged, as did the CCS values of the two most compact conformers (Figure S5 A). The dominant conformer (peak 1), however, broadened and shifted toward larger CCS values, indicating that collisional heating in the gas phase induces a modest expansion of the structure (∼36 Å^2^ increase) (Figure S5). At 20 V, the two compact conformers were no longer detectable, suggesting their conversion into higher-CCS structures that overlap with the broadened main conformer (Figure S5). Base loss, the earliest fragmentation pathway in RNA, was first detected at collision energies of ≥20 V but accounted for less than 0.6% of the precursor ion abundance at 20 V regardless of whether quadrupole isolation was used (Figure S6), confirming that the conformational changes observed at collision energies ≤20 V reflect gas-phase unfolding rather than fragmentation. The peak corresponding to the dominant conformer continued to increase in CCS and width, reflecting the formation of an unresolved ensemble of more extended structures (Figure S5). Interestingly, at 40 V, this trend partially reversed, with a slight compaction observed (Figure S5). However, the CCS of this conformer family remained substantially larger than at 0 V, indicating that the structures populated at high collision energy differ from those present in solution. At even higher collision energies (45–50 V), pronounced fragmentation of the M^8–^ ion was observed, suggesting that the apparent compaction at 40 V may precede collision-induced bond cleavage (Figure S6). Such gas-phase compaction likely reflects disruption of base-pairing interactions, which in turn facilitates fragmentation through loss of neutral nucleobases.

Together, these findings strongly support the conclusion that the compact conformers observed during thermal unfolding reflect genuine solution-phase species rather than artifacts of gas-phase rearrangements. Moreover, due to the high GC content of the riboswitch and the associated strong noncovalent interactions,^59,60^ the M^8–^ ion undergoes fragmentation at elevated collision energies (45–50 V) prior to extensive unfolding (Figures S5 and S6).

## Conclusion

This study highlights the power of integrating temperature-variable native electrospray ionization mass spectrometry, ion mobility spectrometry, ^1^H NMR spectroscopy, and UV absorption to unravel the thermal unfolding behavior of structured RNA. By tracking average charge states across a temperature gradient and combining these results with ^1^H NMR data, we revealed a reproducible triphasic unfolding profile across three distinct temperature regions: early local melting of the upper stem (Region I, 25–55 °C), global loss of secondary structure (Region II, 60–75 °C), and the unexpected formation of compact, non-native phosphate shielded conformers at elevated temperatures (Region III, 80–90 °C).

Ion mobility measurements enabled conformer-resolved structural insight, showing that while most charge states exhibited minimal temperature dependence, the M^9–^ ion displayed slight expansion, and notably, the majority of the M^8–^ ion population underwent marked heat-induced compaction to form two new compact structures. These compact conformers emerged exclusively in this intermediate charge state, consistent with the view that such states best preserve solution-like conformations in the gas phase. Crucially, collision-induced unfolding experiments confirmed that the heat-induced compaction is not related to gas-phase collapse but arises from genuine solution-phase intermediates. The opposing behavior observed during CIU, namely CCS expansion, underscores that the observed structural compaction arises from thermally driven conformational changes occurring in solution, rather than being artifacts of gas-phase activation.

Our findings demonstrate that this integrative approach uniquely captures coexisting RNA conformers even under conditions approaching the boiling point of water, where traditional methods provide limited or no structural information. The ability to detect subtle and unexpected conformational transitions, such as heat-induced compaction, positions this integrative approach as a powerful tool for probing RNA conformational changes under thermal stress. This work reveals that RNA melting is not a one-way route to disorder but can culminate in the formation of compact, non-native ensembles. Recognizing such states is essential for understanding RNA behavior in extreme environments and predicting its resilience in a warming world.

## Supplementary Material



## Notes

(Figure S1) Cytochrome c melting curve; (Figure S2) RNA melting temperature via UV–Vis; (Figure S3) linear calibration curve for calculating CCS; (Figure S4) expanded view of unassigned peak at 90 °C; (Figure S5) CIU analysis for M^8–^ ions; (Figure S6) fragmentation spectra for M^8–^ ions; (Figure S7–S9) arrival-time distributions of M^8–^ ions (Experiments 1–3); (Figure S10–S14) variable-temperature ion mobility heatmaps and arrival-time distributions for M^6–^ to M^17–^ ions (DOCX)
