# Supplementary material for: Uncovering the Structural Landscape of Heat-Induced Riboswitch RNA Unfolding Using Native Variable-Temperature ESI Ion Mobility Mass Spectrometry and 1H NMR
Source: JACS Au. 2026 Jun 19;6(7):3703–12. doi: 10.1021/jacsau.6c00267 (PMC13417269; doi:10.1021/jacsau.6c00267)
Supplement: Supplementary file 1 [file au6c00267_si_001.docx]

**Supplementary Information**

**For**

**Uncovering the Structural Landscape of Heat-Induced Riboswitch RNA Unfolding Using Native Variable-Temperature ESI Ion Mobility Mass Spectrometry and ^1^H NMR**

Sarah V. Heel-Juen^1^, Raphael Plangger^2^, Casey J. Chen^1^, Zachary M. Miller^1^, Fabian Juen^2^, Christoph Kreutz^2^, and Evan R. Williams^1^*

1. Department of Chemistry, University of California, Berkeley, California 94720-1460, USA
2. Institute of Organic Chemistry and Center for Molecular Biosciences Innsbruck (CMBI), University of Innsbruck, Innrain 80/82, 6020 Innsbruck, (Austria)

***Corresponding author: erw@berkeley.edu**

**CONTENTS**

**Figure S1**. Melting curve of Cytochrome C at pH 7.0...............................................S3
**Figure S2.** RNA melting curves measured via UV–Vis absorbance.........................S4
**Figure S3**. Calibration curve for converting drift times to CCS.................................S5
**Figure S4.** Expanded view of the m/z 732.1–736.3 region of the MS spectra at 90°C (Figure 1A).................................................................................................................................................S6
**Figure S5.** Collision-induced unfolding analysis of the riboswitch aptamer for the M⁸⁻ ion..................................................................................................................................................S8
**Figure S6.** Mass spectra of the riboswitch aptamer (z = 8−) recorded at selected collision energies........................................................................................................................................S10

**Figure S7.** Arrival-time distributions of M^8⁻^ ions measured at different temperatures (Experiment 1)..................................................................................................................................................S12

**Figure S8.** Arrival-time distributions of M^8⁻^ ions measured at different temperatures (Experiment 2)..................................................................................................................................................S14
**Figure S9.** Arrival-time distributions of M^8⁻^ ions measured at different temperatures (Experiment 3)……………………………………………………………………………………..................S16
**Figure S10.** Variable-temperature ion mobility heat maps and arrival time distributions for M^6-^ and M^7-^ Ions.................................................................................................................................S17
**Figure S11.** Variable-temperature ion mobility heat maps and arrival time distributions for M^10-^ and M^11-^ Ions……………………...…………………………………………………….............S18
**Figure S12.** Variable-temperature ion mobility heat maps and arrival time distributions for M^12-^ and M^13-^ Ions……………………...…………………………………………………….............S19
**Figure S13.** Variable-temperature ion mobility heat maps and arrival time distributions for M^14-^ and M^15-^ Ions……………………...…………………………………………………….............S20
**Figure S14.** Variable-temperature ion mobility heat maps and arrival time distributions for M^16-^ and M^17-^ Ions……………………...…………………………………………………….............S21


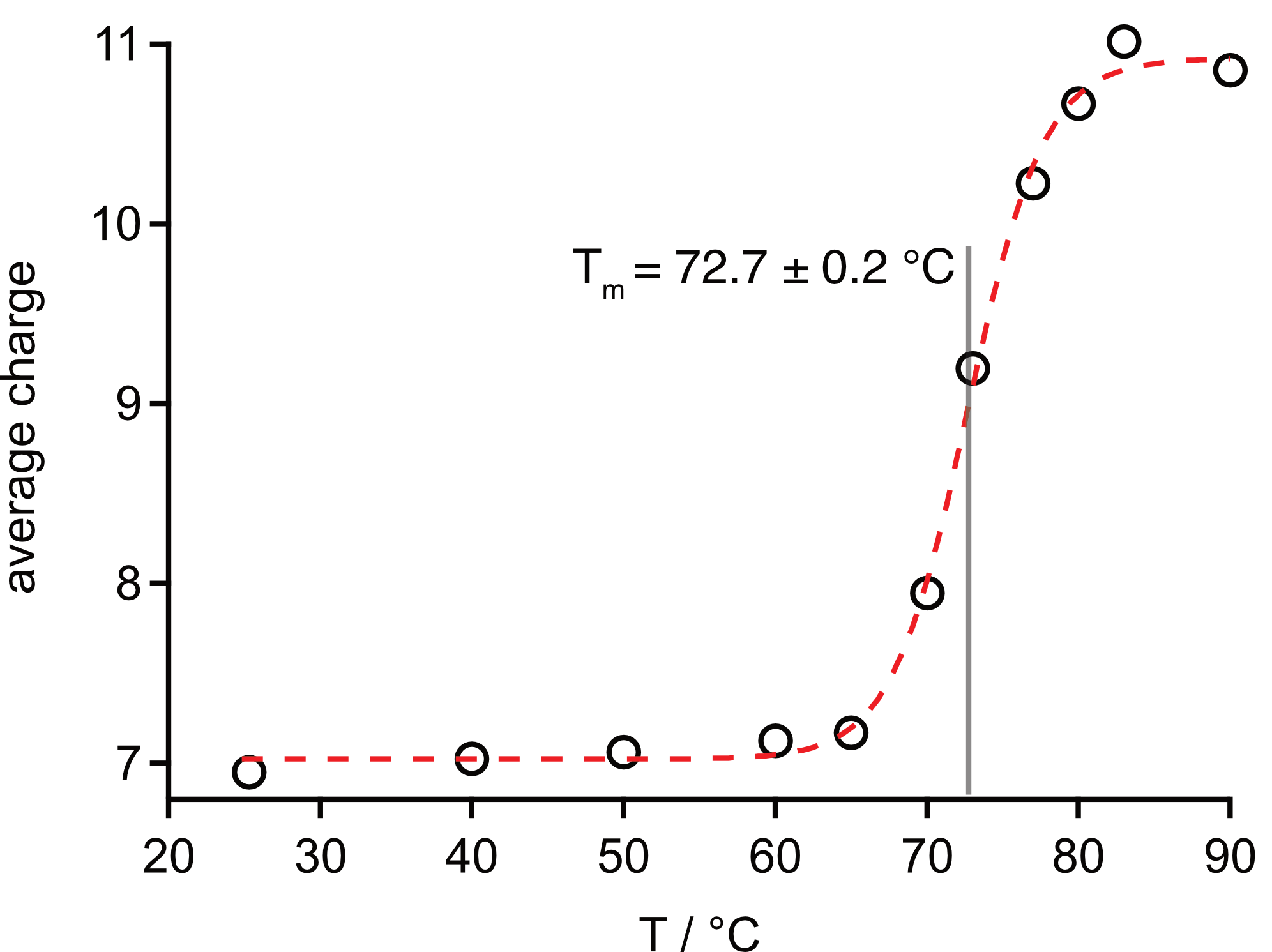


**Figure S1.** Melting curve of Cytochrome *c* at pH 7.0, based on average charge states determined from nanoESI mass spectra at varying solution temperatures. A two-state model fit yields a melting temperature, T_m_, of 72.7 ± 0.2 °C.


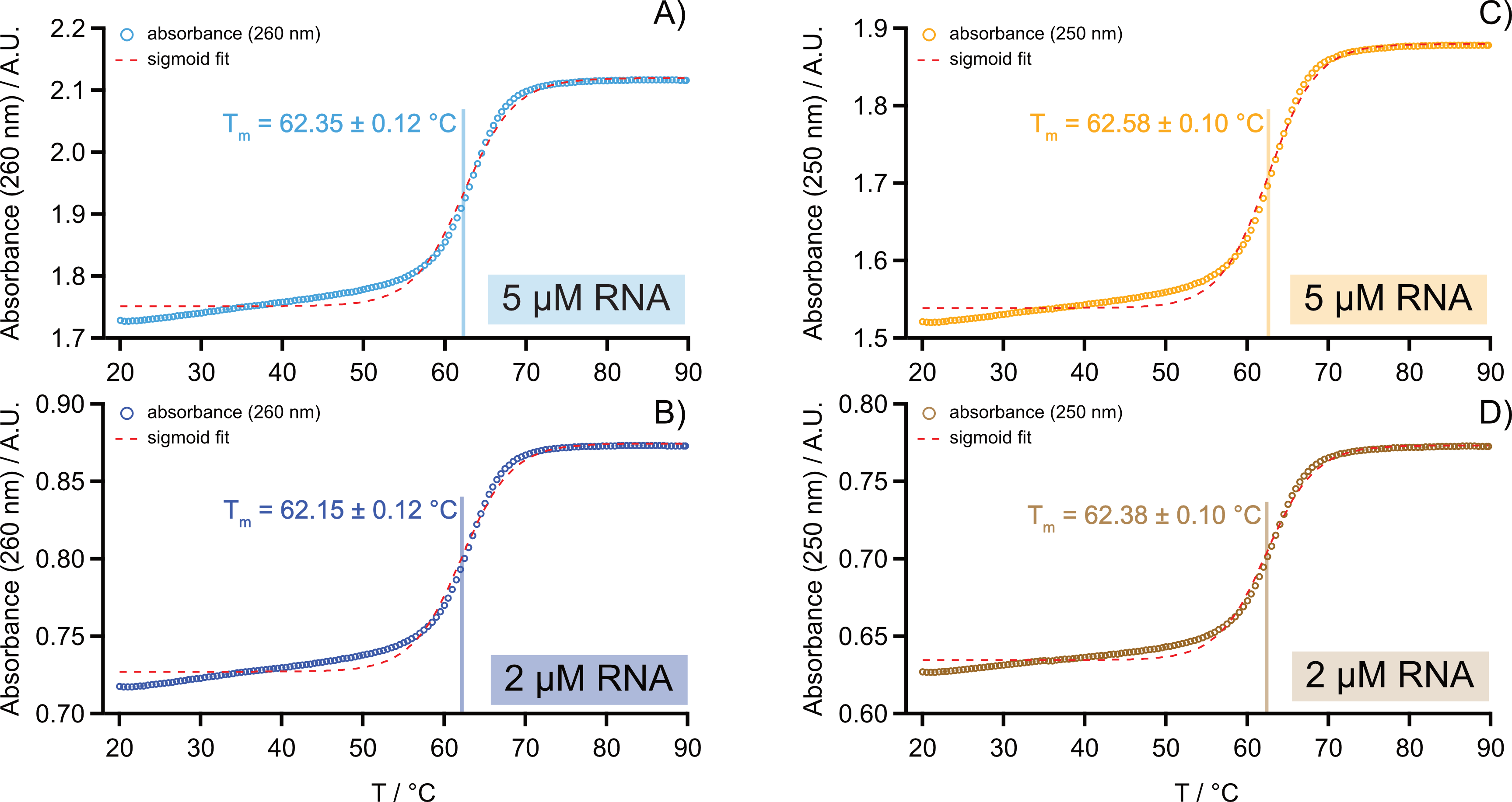


**Figure S2.** RNA melting curves measured via UV absorbance. Sigmoidal fits yield melting temperatures (Tₘ) and corresponding errors are derived from fitting parameters. A-B) Absorbance at 260 nm as a function of temperature for 5 µM and 2 µM RNA concentrations, respectively. C-D) Absorbance at 250 nm as a function of temperature for 5 µM and 2 µM RNA concentrations, respectively


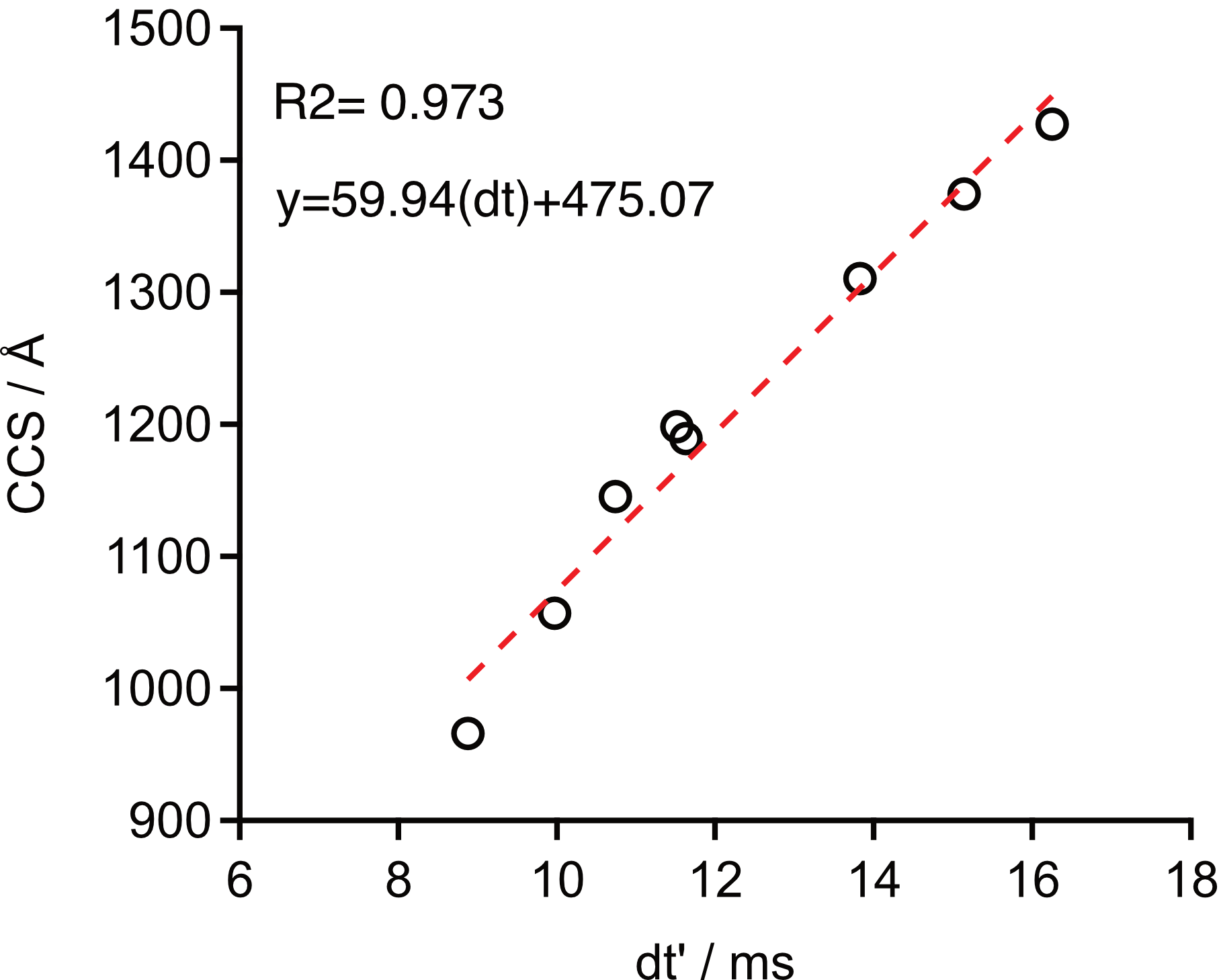


**Figure S3.** Calibration curve for converting drift times to collision cross sections (CCS). EDC (enhanced duty cycle delay coefficient)–corrected drift times (dt′) are plotted against the corresponding calculated helium CCS values (CCS′) for a set of calibrant ions measured in nitrogen. A linear fit to the data yields an R² of 0.973.


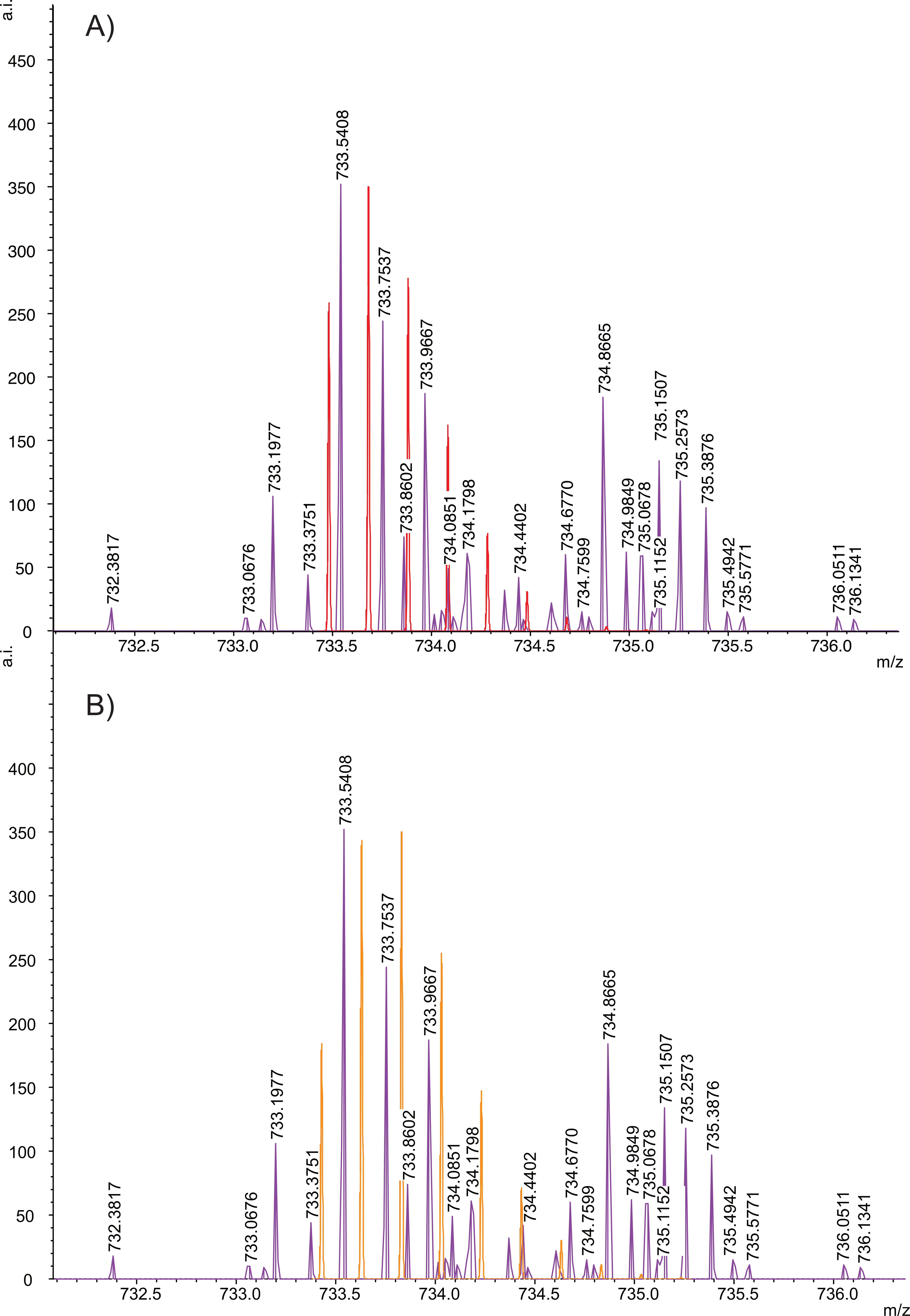


**Figure S4.** Expanded view of the *m*/*z* 732.1–736.3 region of the native nanoESI mass spectrum of the riboswitch aptamer acquired at 90 °C, corresponding to the low-abundance species marked with an asterisk (*) in Figure 1A. **A)** Experimental spectrum (purple) overlaid with a simulated isotope pattern (red) for the terminal *w-*11 RNA fragment (C_106_H_132_N_44_O_80_P_12_, average mass 3674.138 Da, *z* = 5−). **B)** Experimental spectrum (purple) overlaid with the simulated isotope pattern (orange) for PEG-83 (C_166_H_334_O_84_, average mass 3674.414 Da, *z* = 5−). Neither assignment can be confirmed unambiguously. The species appears exclusively at 90 °C and its charge state could not be determined with certainty. The peak is therefore left unassigned; its presence does not affect the interpretation of the charge-state distributions or ion mobility data presented in this study.


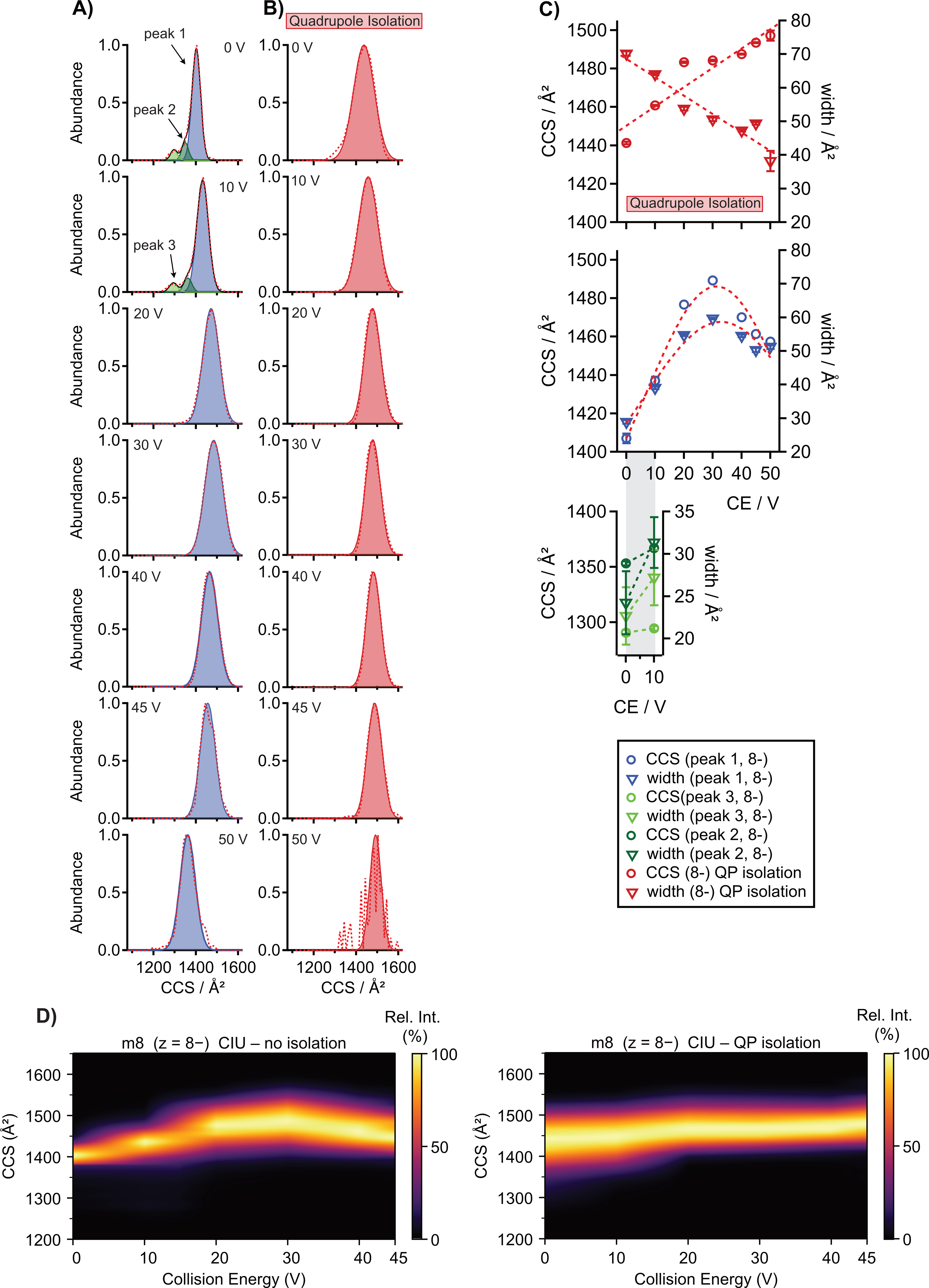


**Figure S5.** Collision-induced unfolding analysis of the riboswitch aptamer for the M⁸⁻ ion. **A–B**) Arrival time distributions recorded at increasing collision energies without (A, blue) and with (B, red) quadrupole (QP) isolation. **C**) Collision cross section (CCS, left axis) and peak width (right axis) of the native conformer plotted as a function of collision energy. Circles and triangles represent CCS and peak width, respectively. Data obtained without QP isolation are shown in blue, compact conformer species - which also increase upon heating in RNA melting experiments - are highlighted in dark and light green. Data acquired with QP isolation are shown in red. **D**) CIU heat map without quadrupole isolation (left) and CIU heat map with QP isolation (right) of the M^8-^ ion. In both panels, relative ion mobility intensity is shown as a function of CCS (Å^2^, *y*-axis) and collision energy (CE, V, *x*-axis), normalized to 100% per collision energy step. Data included into CIU heat maps ranging from CE = 0–45 V; data at 50 V were not included into heat maps due to insufficient precursor signal intensity because of fragmentation at elevated activation energies (see Figure S6). Minor compact conformer populations identified in the arrival time distributions (panel A) are not very well resolved in the heat map representation due to their low relative abundance (~5–8%).


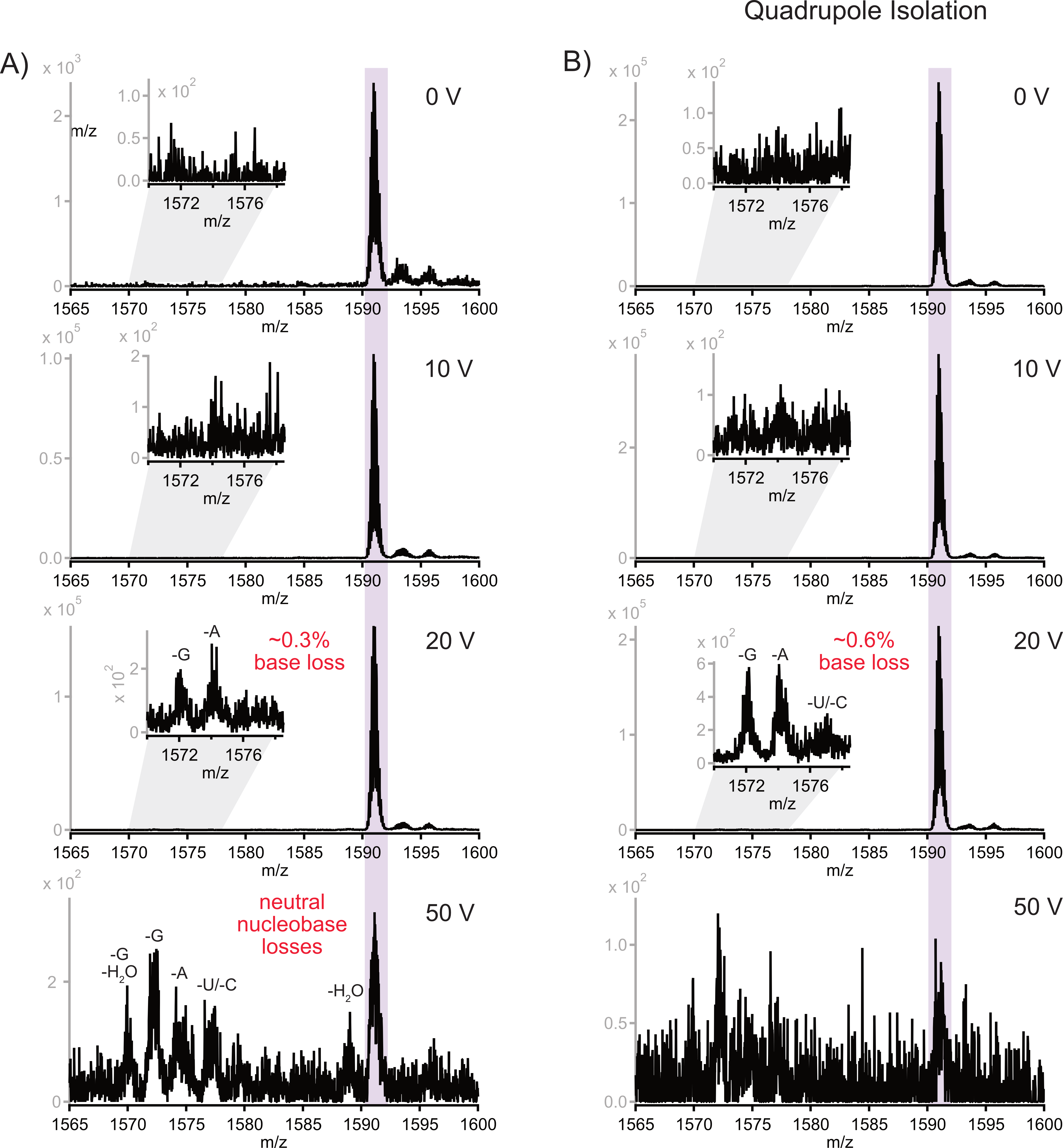


**Figure S6.** Mass spectra of the M⁸⁻ precursor ion region (*m*/*z* 1565–1600) of the riboswitch aptamer recorded at selected collision energies, illustrating the onset and extent of collision-induced nucleobase loss, the predominant low-energy fragmentation pathway in RNA ions. Insets show an expanded view of the neutral base loss region (*m*/*z* 1570–1578.5). **A)** Mass spectra recorded without quadrupole (QP) isolation at CE = 0, 10, 20 and 50 V. At CE = 0 V, a single dominant precursor ion is observed at *m*/*z* ~1591 (*z* = 8−), with minor compact conformer populations visible as a low-intensity shoulder toward lower *m*/*z* (peaks 2 and 3; Figure S5A). At CE = 10 V, no significant fragment ions corresponding to base loss are detected. At CE = 20 V, minor neutral base losses (~0.3% relative to precursor) are first observed, marking the onset of fragmentation. The disappearance of the compact conformer populations (peaks 2 and 3) at CE ≥ 20 V therefore very likely reflects gas-phase conformational transitions of the dominant conformer (peak 1) rather than fragmentation at 20 V. At CE = 50 V, neutral nucleobase losses become clearly visible (−G & −H_2_O, −G, −A, −U/−C, −H_2_O). **B**) Mass spectra recorded with quadrupole isolation at CE = 0, 10, 20 and 50 V. At CE = 0 V and CE = 10 V, no significant fragment ions are detected. At CE = 20 V, minor neutral base losses (~0.6% relative to precursor) are first observed (−G, −A, −U/−C), marking the onset of fragmentation. At CE = 50 V, the precursor signal is largely depleted, and the spectrum is dominated by fragment ions. These data confirm that CIU heat maps (Figure S5) are reported only up to CE = 45 V.

**
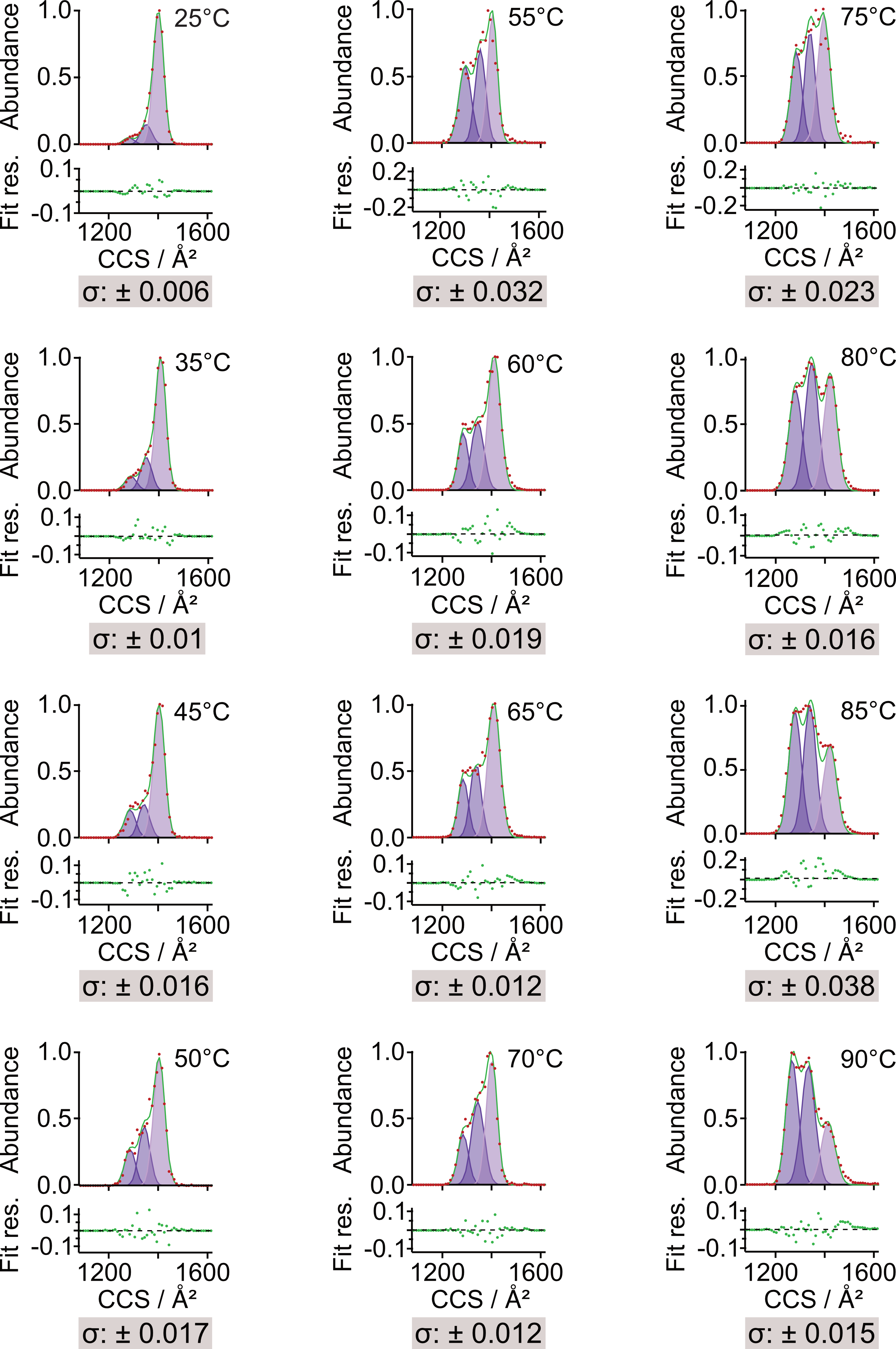
**

**Figure S7 (Experiment 1).** Arrival-time distributions of M^8−^ ions measured at different temperatures. Experimental data are shown as red dots. The total fit is a triple-Gaussian model plotted as a green line; the individual Gaussian components are shaded, with the room-temperature conformer shown in light purple and compact conformers observed at higher temperatures shown in dark purple. The y-axis is normalized to 0–1. Fit residuals are shown beneath each distribution. Fit quality is quantified by σ, defined as the root-mean-square (RMS) of the residuals reported by Igor Pro (Fit Wave Statistics).


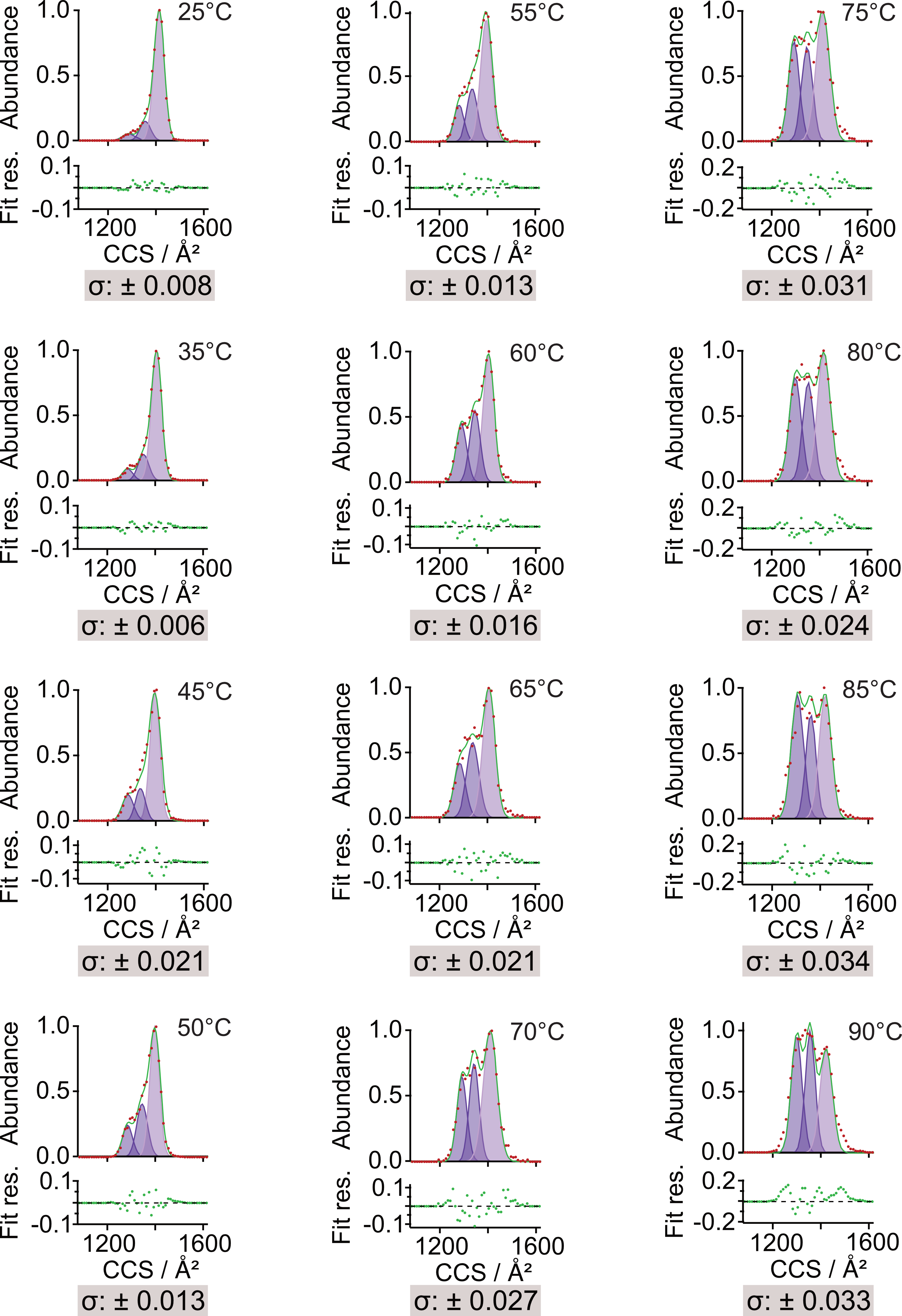


**Figure S8 (Experiment 2).** Arrival-time distributions of M^8−^ ions measured at different temperatures. Experimental data are shown as red dots. The total fit is a triple-Gaussian model plotted as a green line; the individual Gaussian components are shaded, with the room-temperature conformer shown in light purple and compact conformers observed at higher temperatures shown in dark purple. The *y*-axis is normalized to 0–1. Fit residuals are shown beneath each distribution. Fit quality is quantified by σ, defined as the root-mean-square (RMS) of the residuals reported by Igor Pro (Fit Wave Statistics).


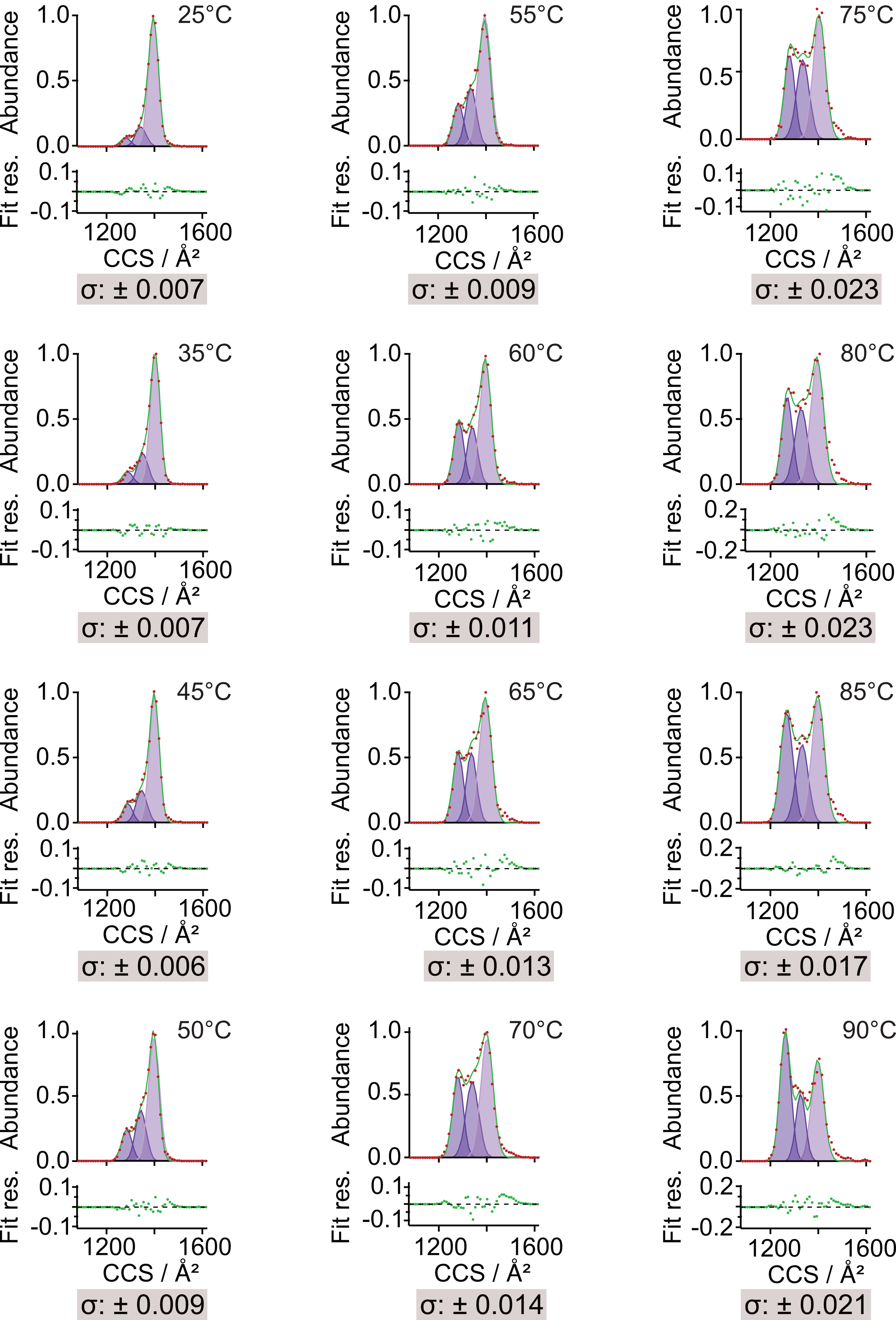


**Figure S9 (Experiment 3).** Arrival-time distributions of M^8−^ ions measured at different temperatures. Experimental data are shown as red dots. The total fit is a triple-Gaussian model plotted as a green line; the individual Gaussian components are shaded, with the room-temperature conformer shown in light purple and compact conformers observed at higher temperatures shown in dark purple. The *y*-axis is normalized to 0–1. Fit residuals are shown beneath each distribution. Fit quality is quantified by σ, defined as the root-mean-square (RMS) of the residuals reported by Igor Pro (Fit Wave Statistics).


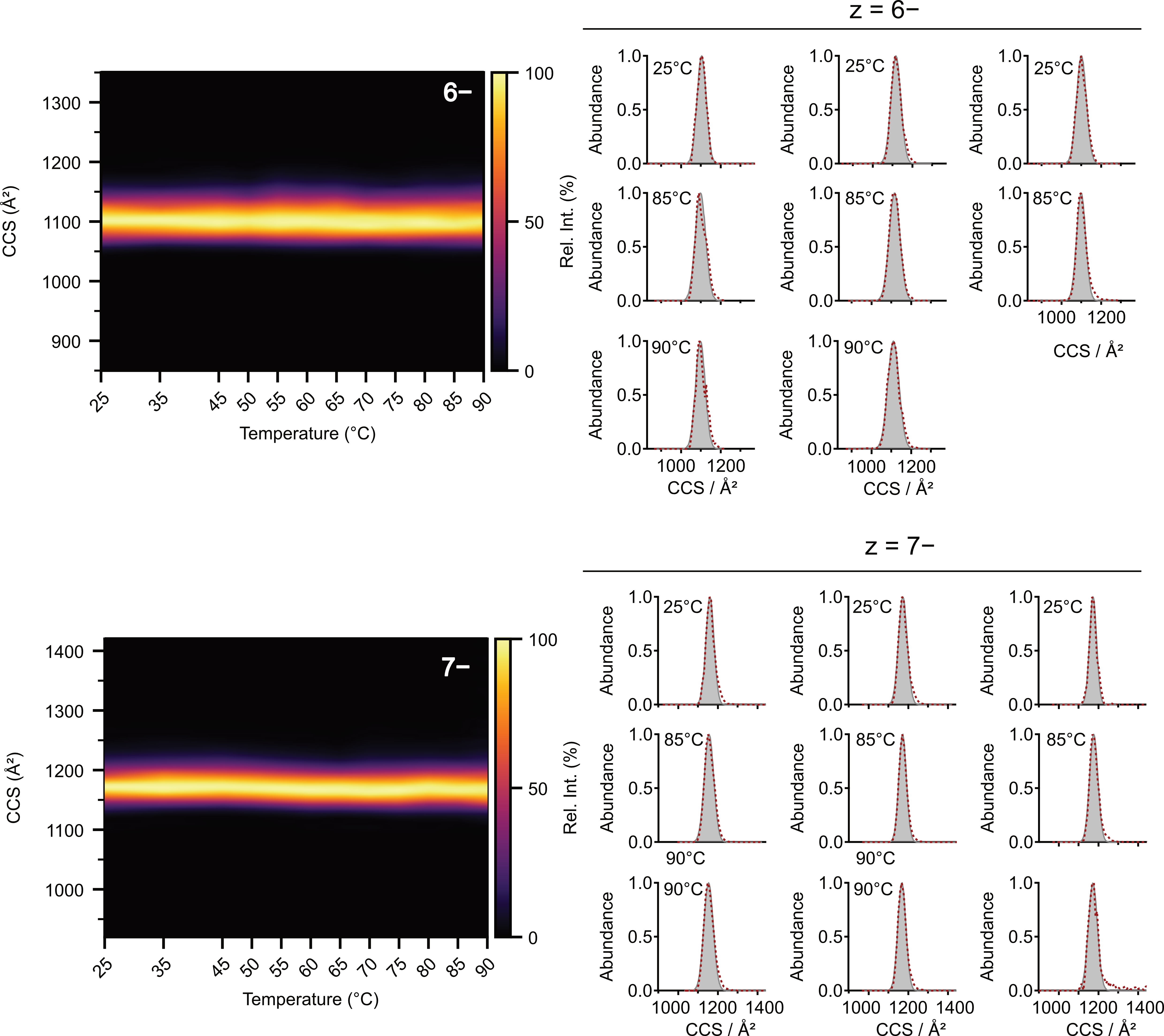


**Figure S10.** Variable-temperature ion mobility heat maps and arrival time distributions for charge states *z* = 6− and *z* = 7−. Left panels show 2D heat maps with relative intensity encoded by color (inferno scale, 0–100%, normalized per temperature step) as a function of collision cross section (CCS) and solution temperature (25–90 °C) representing the average of *n* = 2–3 independent measurements. Right panels show arrival time distributions (ATDs) at selected temperatures (25, 85, and 90 °C) for three independent measurements.


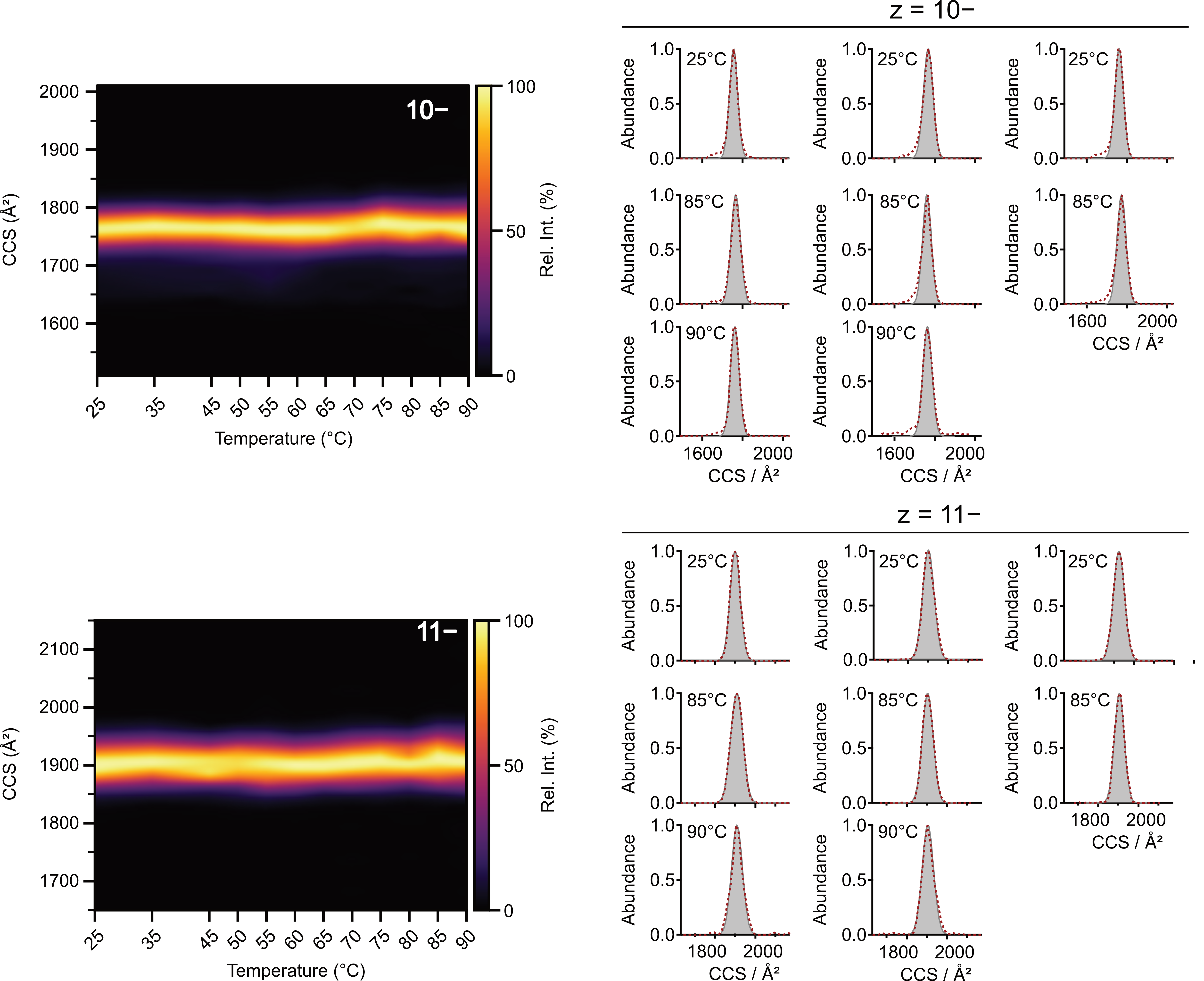


**Figure S11.** Variable-temperature ion mobility heat maps and arrival time distributions for charge states *z* = 10− and *z* = 11−. Left panels show 2D heat maps with relative intensity encoded by color (inferno scale, 0–100%, normalized per temperature step) as a function of collision cross section (CCS) and solution temperature (25–90 °C) representing the average of *n* = 2–3 independent measurements. Right panels show arrival time distributions (ATDs) at selected temperatures (25, 85, and 90 °C) for three independent measurements.


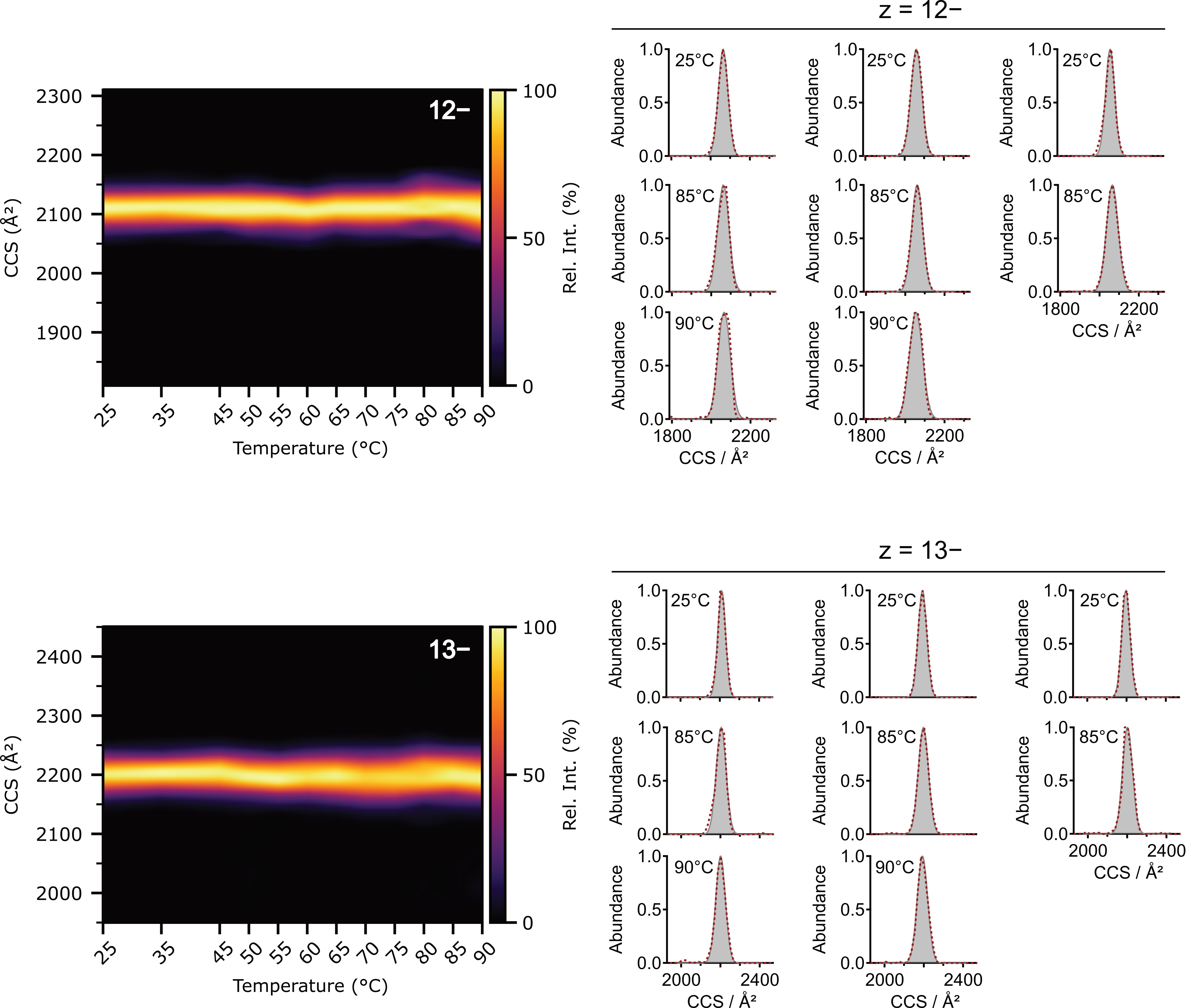


**Figure S12.** Variable-temperature ion mobility heat maps and arrival time distributions for charge states *z* = 12− and *z* = 13−. Left panels show 2D heat maps with relative intensity encoded by color (inferno scale, 0–100%, normalized per temperature step) as a function of collision cross section (CCS) and solution temperature (25–90 °C) representing the average of *n* = 2–3 independent measurements. Right panels show arrival time distributions (ATDs) at selected temperatures (25, 85, and 90 °C) for three independent measurements.


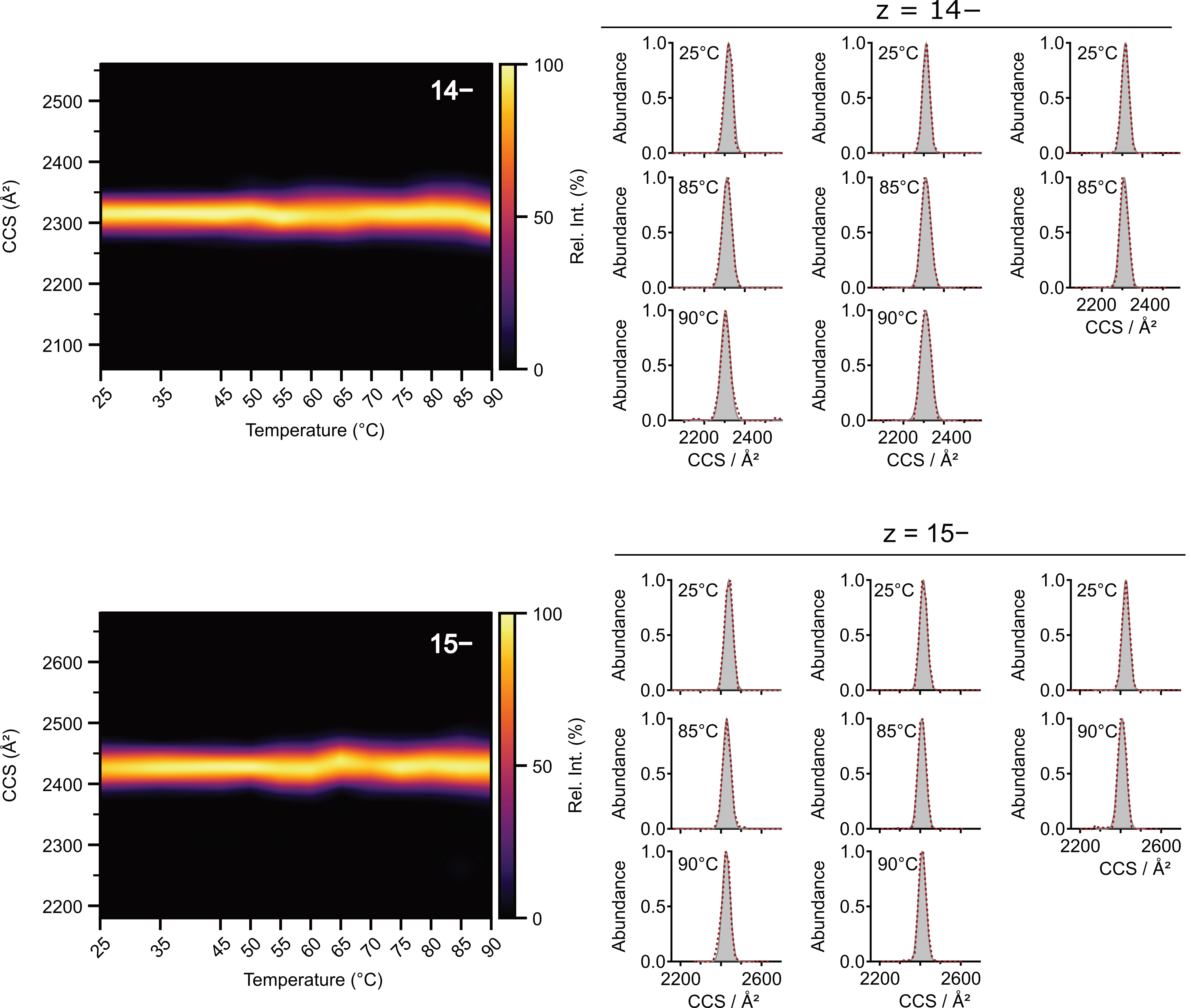


**Figure S13.** Variable-temperature ion mobility heat maps and arrival time distributions for charge states *z* = 14− and *z* = 15−. Left panels show 2D heat maps with relative intensity encoded by color (inferno scale, 0–100%, normalized per temperature step) as a function of collision cross section (CCS) and solution temperature (25–90 °C) representing the average of *n* = 2–3 independent measurements. Right panels show arrival time distributions (ATDs) at selected temperatures (25, 85, and 90 °C) for three independent measurements.


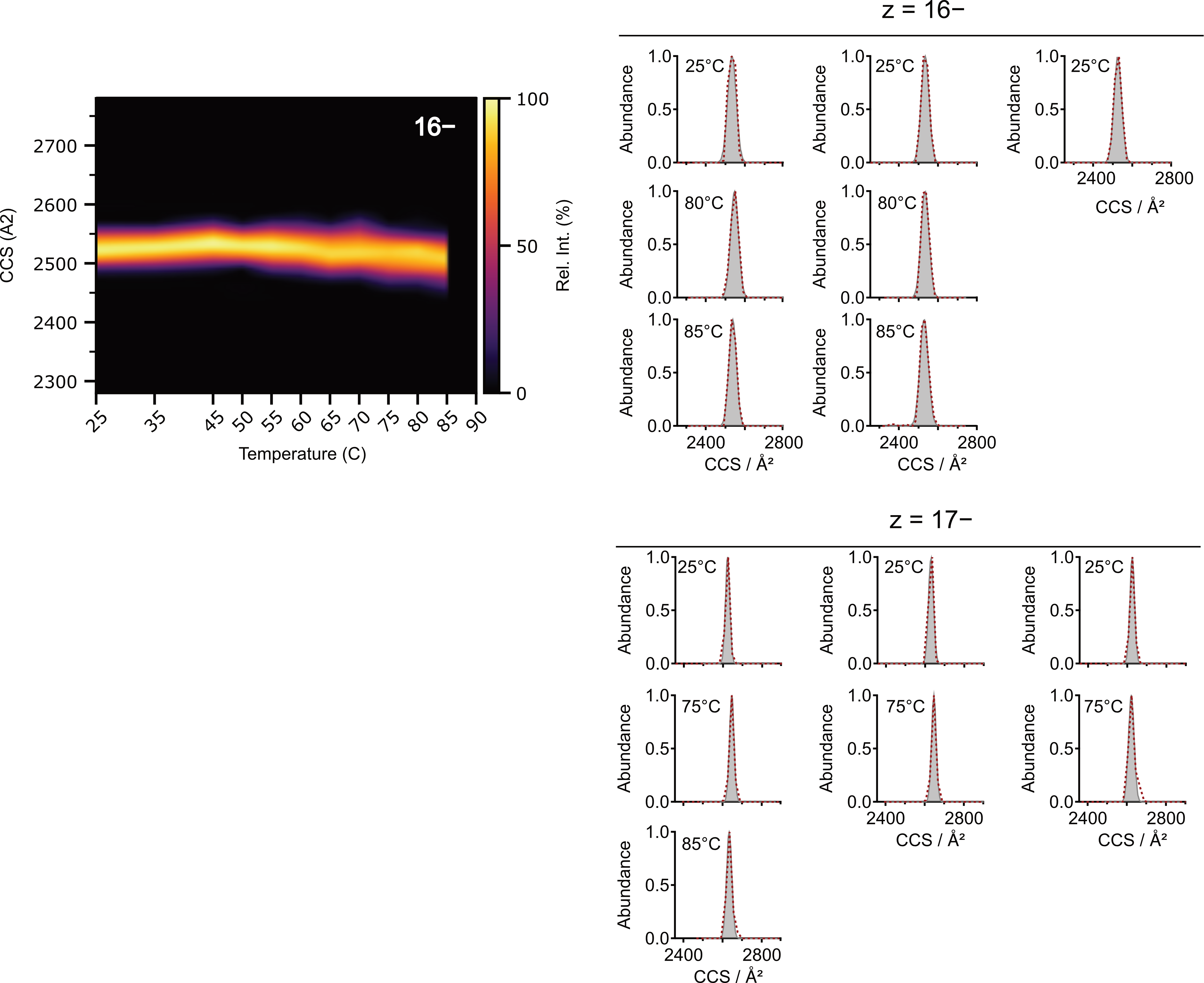


**Figure S14.** Variable-temperature ion mobility heat maps and arrival time distributions for charge states *z* = 16− and *z* = 17−. Left panels show 2D heat maps with relative intensity encoded by color (inferno scale, 0–100%, normalized per temperature step) as a function of collision cross section (CCS) and solution temperature (25–90 °C), representing the average of *n* = 1–3 independent measurements. Right panels show arrival time distributions (ATDs) at selected temperatures for three independent measurements. For *z* = 16−, data are shown up to 85 °C as no signal was detected at 90 °C. For *z* = 17−, this charge state was only sporadically populated across the temperature range and independent experiments (Figure 2-A), resulting in insufficient temperature coverage for reliable interpolation between individual temperature steps; no heatmap was therefore generated for this charge state. Right panels show arrival time distributions (ATDs) at selected temperatures for three independent measurements shown where data were available.
